# An individual-based modelling approach to estimate landscape connectivity for bighorn sheep (*Ovis canadensis)*

**DOI:** 10.7717/peerj.2001

**Published:** 2016-05-05

**Authors:** Corrie H. Allen, Lael Parrott, Catherine Kyle

**Affiliations:** Okanagan Institute for Biodiversity, Resilience, and Ecosystem Services (BRAES), University of British Columbia, Kelowna, BC, Canada

**Keywords:** Wildlife corridors, Habitat connectivity, Agent-based model, Range shifts, Biological conservation, Ecosystem management

## Abstract

**Background**. Preserving connectivity, or the ability of a landscape to support species movement, is among the most commonly recommended strategies to reduce the negative effects of climate change and human land use development on species. Connectivity analyses have traditionally used a corridor-based approach and rely heavily on least cost path modeling and circuit theory to delineate corridors. Individual-based models are gaining popularity as a potentially more ecologically realistic method of estimating landscape connectivity. However, this remains a relatively unexplored approach. We sought to explore the utility of a simple, individual-based model as a land-use management support tool in identifying and implementing landscape connectivity.

**Methods**. We created an individual-based model of bighorn sheep *(Ovis canadensis)* that simulates a bighorn sheep traversing a landscape by following simple movement rules. The model was calibrated for bighorn sheep in the Okanagan Valley, British Columbia, Canada, a region containing isolated herds that are vital to conservation of the species in its northern range. Simulations were run to determine baseline connectivity between subpopulations in the study area. We then applied the model to explore two land management scenarios on simulated connectivity: restoring natural fire regimes and identifying appropriate sites for interventions that would increase road permeability for bighorn sheep.

**Results.** This model suggests there are no continuous areas of good habitat between current subpopulations of sheep in the study area; however, a series of stepping-stones or circuitous routes could facilitate movement between subpopulations and into currently unoccupied, yet suitable, bighorn habitat. Restoring natural fire regimes or mimicking fire with prescribed burns and tree removal could considerably increase bighorn connectivity in this area. Moreover, several key road crossing sites that could benefit from wildlife overpasses were identified.

**Discussion.** By linking individual-scale movement rules to landscape-scale outcomes, our individual-based model of bighorn sheep allows for the exploration of how on-the-ground management or conservation scenarios may increase functional connectivity for the species in the study area. More generally, this study highlights the usefulness of individual-based models to identify how a species makes broad use of a landscape for movement. Application of this approach can provide effective quantitative support for decision makers seeking to incorporate wildlife conservation and connectivity into land use planning.

## Introduction

Maintaining landscape connectivity, the ability of a landscape to facilitate movement between areas of good habitat (Taylor et al., 1993; Tischendorf & Fahrig, 2000; Fischer & Lindenmayer, 2007; Beier et al., 2011), has become a central priority in conservation (Heller & Zavaleta, 2009). Connectivity is widely recognized as an important component of ecologically functional landscapes. Connections between areas of good habitat or subpopulations of a species can support key biological processes such as upholding natural animal movement between foraging, breeding, and migrating sites; decreasing genetic isolation (Keyghobadi, 2007); and promoting colonization into new habitats (Gustafson & Gardner, 1996). More broadly, connected habitats are an important component of functioning ecosystems and can support provisioning of ecosystem services, which in turn increases the socio-economic value of natural areas (Biggs et al., 2012; Mitchell et al., 2015). Connectivity conservation has added urgency, as habitat fragmentation is likely to exacerbate climate change pressures on species (Opdam & Wascher, 2004).

Preserving linkages for dispersing individuals across a landscape with corridors and/or stepping-stones is a compelling strategy to create a functionally connected network in place of disjoint habitat patches. Corridors are traditionally conceptualized as swaths of a landscape that connect otherwise isolated patches of habitat. Although the degree to which species use corridors differs across taxa, in general, species react positively to corridors (Gilbert-Norton et al., 2010). Numerous spatial analyses have been developed to estimate where corridors are likely located on a given landscape. An ongoing challenge however, is to integrate species-specific movement behaviour and a realistic representation of how species perceive their landscape into methods used to identify corridors. Methods to estimate locations of wildlife corridors largely employ resistance-based models to predict which components of a landscape facilitate movement for a species of interest (Zeller, McGarigal & Whiteley, 2012). For example, two popular tools to identify corridors are least cost path analysis (Adriaensen et al., 2003) and circuit theory (McRae et al., 2008). Both of these approaches simulate a landscape as a resistance to movement layer wherein each cell within a rasterized landscape is given a value representing the ecological cost incurred by an individual moving across that cell (Spear et al., 2010). Path-finding algorithms will then identify optimal routes across the resistance layer. The least cost path approach is readily accessible both to researchers and field ecologists, which is probably a driving factor in its wide application (Epps et al., 2007; Driezen et al., 2007; Creech et al., 2014). This approach requires modellers to identify locations on the landscape to connect and a single, best route between these locations is then identified. Despite the usefulness of least cost path simulations, this approach implicitly assumes that individuals have complete knowledge of their landscape, which is a somewhat erroneous assumption (Fahrig, 2007). In contrast to least cost path, circuit theory draws on the resemblance between random walks observed in natural systems and the flow of electrical current through a circuit (Cowley, Johnson & Pocock, 2015; McRae et al., 2008). This approach assumes that individuals have no prior knowledge of their landscape extending past adjacent pixels and the resulting maps will consider many possible routes across the landscape (McClure, Hansen & Inman, in press; Walpole et al., 2012). Moreover, circuit theory does not require pre-defined start and destination points on the landscape to connect as an input (Pelletier et al., 2014).

There has been growing interest in better integrating individual behaviour, perceptual range, and movement decisions into corridor models to enhance the ecological realism of connectivity design (Sawyer, Epps & Brashares, 2011). Individual-based models are emerging as a promising tool to assess, plan, and implement landscape connectivity (Baguette & Van Dyck, 2007; Beier et al., 2011; Kool, Moilanen & Treml, 2013). In general, individual (or agent) based models (hereafter IBM) create agents that interact with each other and/or their environment through prescriptive rules (Grimm et al., 2005). Emergent system level patterns then arise from these local interactions. Individual-based models have been widely applied in ecology as a simulation tool to explore systems where individual, localized decisions play into system-level dynamics. This is a potentially powerful approach to explore landscape connectivity because researchers can simulate individuals responding to local environmental conditions to make movement decisions and allow corridors to emerge as a result of animals interacting with their landscape. An IBM therefore may provide a more nuanced estimate of connectivity than least cost path analysis or circuit theory by highlighting all components of a landscape with the potential to facilitate animal movement rather than only the least costly routes or paths of least resistance. This is a potential advantage of IBMs because it results in a gradient of possible locations for conservation actions that can fit with other land use objectives. Individual-based models therefore provide a compelling framework to explore how management actions will impact connectivity before investing in on-the-ground work, which is important given the paucity of resources available in most conservation projects. There are a handful of studies that have used IBMs to explore landscape connectivity both theoretically and experimentally (Graf et al., 2007; Pe’er et al., 2011; Kramer-Schadt et al., 2011; Kanagaraj et al., 2013; Aben et al., 2014). Prior work has demonstrated that IBMs better reflect how animals move across a landscape than least cost path or circuit theory, and thus can provide an improved estimate of connectivity (Coulon et al., 2015). Despite the potential advantages of IBMs over least cost path and circuit theory approaches, this remains a relatively unexplored approach to identify components of a landscape that contribute to connectivity.

The overarching aim of this study is to demonstrate the utility of an IBM as a land-use management support tool to increase species-specific connectivity across a landscape. Bighorn sheep (*Ovis canadensis*) in the Okanagan Valley of British Columbia, Canada, were used for this analysis. Bighorn sheep were once widely distributed in this region, but habitat loss as a result of human and natural land use transformations has led to segregation of the population into three separate subpopulations with major roads, rivers, and other obstacles to movement limiting dispersal ability (Demarchi, Hartwig & Demarchi, 2000). There is interest in maintaining connectivity across this landscape for two purposes. First, a connected landscape could facilitate dispersal between subpopulations of sheep to increase gene flow. Second, maintaining linkages between existing bighorn subpopulations and currently unoccupied yet suitable bighorn sheep habitat could promote colonization into new habitats and range shifts as a response to changing climates and to increasing human disturbance in existing bighorn sheep range. Specific objectives of this work are three-fold: (i) to determine if dispersal is likely to occur between present-day bighorn sheep subpopulations without management intervention; (ii) to highlight how bighorn sheep might make broad use of this landscape to facilitate range expansion into currently unoccupied yet suitable habitat; and (iii) to explore how proposed land-use management options might impact bighorn sheep connectivity.

## Materials & Methods

In the following, we develop an individual-based model to guide land management plans to increase bighorn sheep connectivity in the Okanagan Valley of British Columbia, Canada. For this study, we relax the definition of a corridor to include any component of a landscape that promotes movement of individuals, including swaths of good vegetation between habitat patches or small intervening stepping-stones. Our analysis followed three steps. First, to determine if dispersal is likely between subpopulations of sheep and where connectivity gaps are, we constructed an IBM in which virtual bighorn sheep move independently from pre-determined locations across a rasterized landscape according to behavioural movement rules. Bighorn sheep continue to wander until they either leave the landscape, become “stuck”, or the simulation terminates after 2,000 time steps. By aggregating the movement paths of simulated sheep across model iterations, we then identified areas of the landscape contributing to connectivity. Second, to identify how bighorn sheep might make broad use of this landscape to facilitate range shifts or expansions, we adjusted the IBM to start from any pixel with suitable bighorn habitat regardless of whether bighorns currently occupy that area or not. Third, we explored the effectiveness of two proposed management scenarios that aim to facilitate bighorn sheep dispersal in this area: restoring a more natural fire regime to remove densely forested areas and identifying optimal road crossing locations to prioritize for actions that could make roads more permeable to bighorn sheep movement.

### Study area and study species

**Figure 1:**
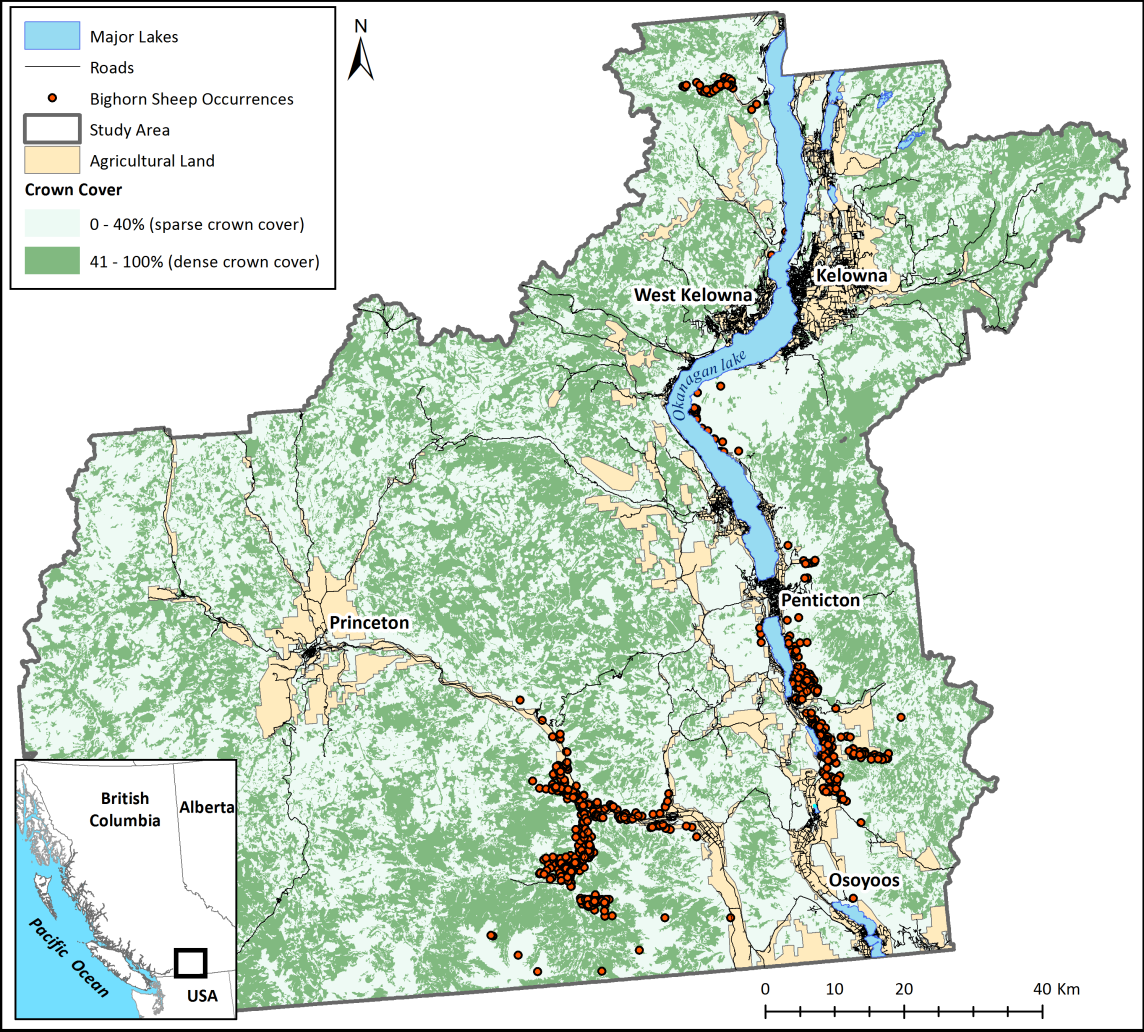
The study area. The main map panel shows major land cover classes and recorded occurrences of bighorn sheep. The insert shows the position of the study area in British Columbia, Canada.

This study was located in the Okanagan Valley of British Columbia, Canada (Fig. 1), a region facing rapid land use and land cover change due to a human population growth rate that is one of the highest in Canada. This region was chosen because it clearly illustrates the need for tools to explore and plan for connectivity. Despite agricultural and urban land cover in the valley bottom, this landscape is unique in that it has considerable remaining habitat to preserve connectedness as human land-use change proceeds. The challenge of determining how and where to preserve corridors however remains. Habitat isolation is regarded as the most pervasive threat to bighorn sheep persistence on the Okanagan landscape (Demarchi, Hartwig & Demarchi, 2000). Despite areas of core habitat remaining largely intact, natural and anthropogenic barriers to movement surround bighorn populations and can several impede dispersal ability. These include wide valleys and plateaus, densely vegetated areas (DeCesare & Pletscher, 2006), roads (Epps et al., 2005), large urban centers (Rubin et al., 2002), and recreation trails that are frequently used by humans and dogs (MacArthur, Johnston & Geist, 1979). Bighorns are listed as “vulnerable” by the Province of British Columbia’s Species at Risk Act and the existing herds are vital to maintaining the species’ persistence. There is a clear need to preserve or restore physical connections between subpopulations along with connections to potential future habitats to facilitate range expansions and shifts.

Bighorn sheep occurrence data was provided by the Provincial Ministry of Forests, Lands, and Natural Resources (years 1968–2013; Fig. 1). Although this data set encompasses sheep occurrences from many years, the location of bighorn sheep has not changed considerably throughout the dataset. Note that this occurrence data was sampled with a bias for areas known to have bighorn sheep rather than an inclusive search of the entire landscape. Absences of bighorn occurrences in Fig. 1 therefore are not necessarily absences of bighorns in the real landscape.

We drew extensively on site-specific government reports supplemented with literature from the greater bighorn range to profile bighorn sheep movement behaviour. The following factors have repeatedly been demonstrated as important to bighorn movement:

(i) Proximity to escape terrain—Escape terrain includes steep and rocky areas that are challenging for predators to traverse. The use of an area decreases as distance to escape terrain increases (DeCesare & Pletscher, 2006; Demarchi, Hartwig & Demarchi, 2000; Shannon et al., 1975; Smith, Flinders & Winn, 1991; Tilton & Willard, 1982; Warman et al., 1998), and
(ii) Preference for sparse vegetation—bighorns depend largely on acute vision to detect threats and therefore avoid densely vegetated areas or areas with a closed canopy (Demarchi, Hartwig & Demarchi, 2000; DeCesare & Pletscher, 2006).

Additionally, roads (Epps et al., 2005; MacArthur, Johnston & Geist, 1979), rivers (Smith, Flinders & Winn, 1991), and large lakes (Demarchi, Hartwig & Demarchi, 2000) are obstacles to bighorn sheep movement. Although sheep habitat selection has been moderately well studied in this region, there exists little information regarding dispersal behaviour; however, work in the broader bighorn range has shown that sheep will, whenever possible, stay close to escape terrain and remain in areas with good visibility. We therefore restricted agent movement to regions that satisfy the movement rules outlined in Table 1 with the understanding that this likely underestimates connectivity by not including dispersal through poor habitat.

**Table 1:** Bighorn sheep movement characteristics.

Movement characteristics	Description	Corresponding rule in the model	References
Close proximity to escape terrain	Steep terrain with interspersed rocky outcrops.	Escape terrain is defined in the model as any slope greater than 40 degrees. Bighorns attempt to remain within 400 m of escape terrain with the likelihood of a sheep travelling further than 400 m from escape terrain decreasing as described by *y* = 188.21*e*^−0.0016*x*^. This equation was derived from a habitat suitability model previously developed for Okanagan bighorn sheep (Warman et al., 1998)	(DeCesare & Pletscher, 2006; Demarchi, Hartwig & Demarchi, 2000; Shannon et al., 1975; Smith, Flinders & Winn, 1991; Tilton & Willard, 1982)
Preference for sparse vegetation	Bighorn sheep avoid densely vegetated areas or areas with a closed canopy	Sheep will not occupy a cell with more than 40% crown cover	(DeCesare & Pletscher, 2006; Demarchi, Hartwig & Demarchi, 2000; Smith, Flinders & Winn, 1991)
Roads	Bighorns sheep are severely deterred by roads, particularly highways and major streets	Sheep cannot occupy a cell with a road	(Epps et al., 2005; MacArthur, Johnston & Geist, 1979)
Ability to cross rivers and lakes	Bighorn sheep rarely cross rivers and never cross large water bodies	Sheep cannot occupy a cell with a lake or river	(Demarchi, Hartwig & Demarchi, 2000)

### Building the individual-based model

The individual-based model presented here is designed to capture critical processes underlying bighorn sheep movement within the Okanagan Valley, British Columbia, Canada to identify where connectivity exists across the landscape. This model was constructed in the Repast Simphony programming environment (North et al., 2013). A complete description of the model following the Objectives, Design concepts and Details (ODD) protocol is included in the Supplemental Information.

### The representation of space

A virtual grid provides a physical environment where sheep agents are located and interact with their landscape. This grid is created with the following land uses: slope in degrees, percent crown cover, lakes, rivers, and roads. Each spatial data layer was collected from government open-data sites (Table S1) and registered within the coordinate system NAD_1983_BC_Environment_Albers. A spatial resolution of 75 m × 75 m was used. This resolution was selected to ensure the spatial scale over which bighorn sheep likely inform movement decisions across their landscape was captured and to allow for fast model execution. Moreover, from a management perspective, this resolution is at a fine enough scale to inform land use decision-making. The landscape is static and shows no diurnal or seasonal variation.

### Bighorn sheep agents

**Figure 2:**
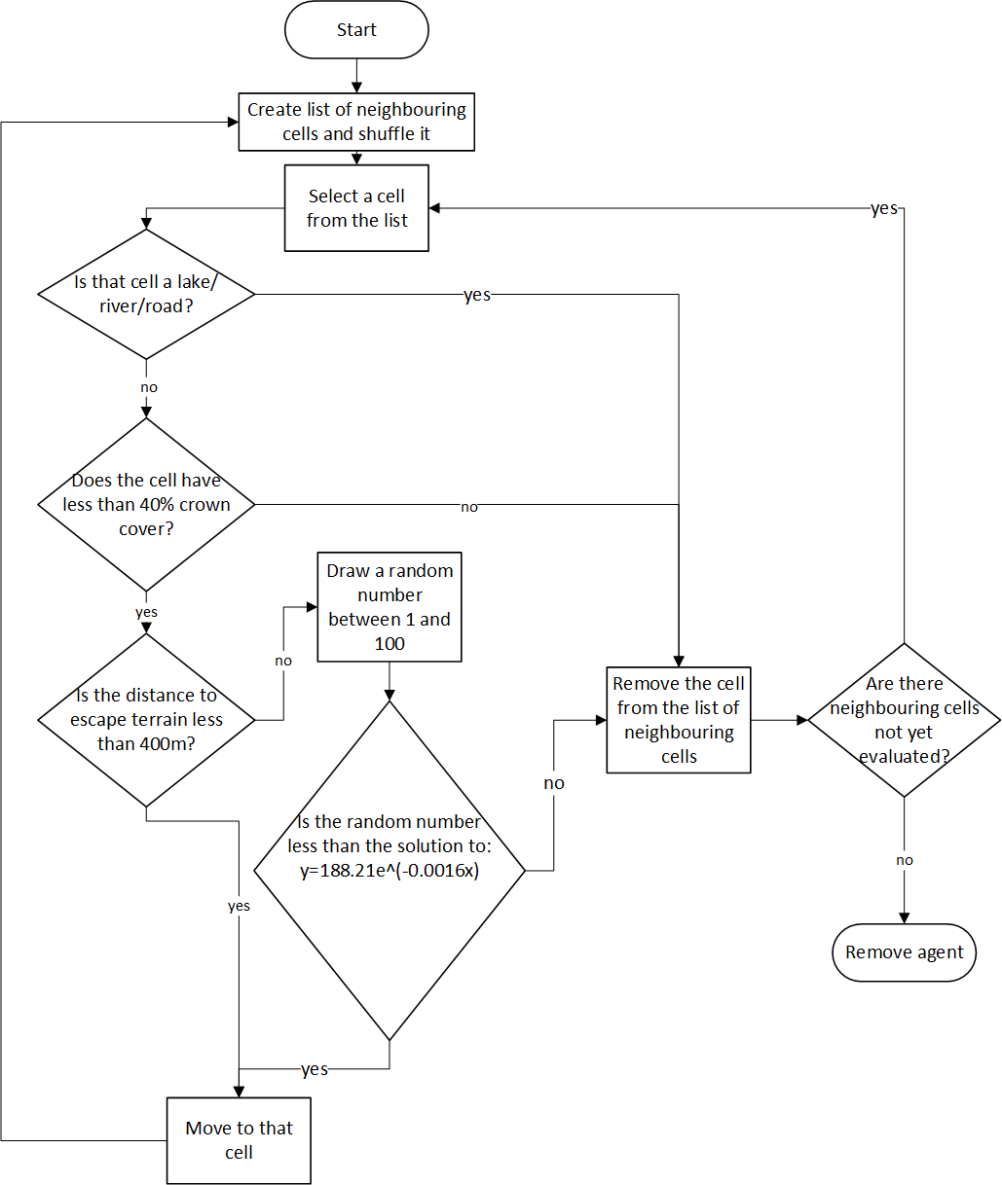
Behaviour rules of the bighorn sheep agents. For the equation used to determine the probability of a bighorn moving to a cell given its distance to escape terrain, *x* is the distance in meters from that cell to escape terrain, and *y* is the resulting probability of moving to that cell.

Bighorn sheep agents are reactive to the accessibility of escape terrain and open vegetation cover that provides visibility. They also react to the presence of barriers to movement including roads, rivers, and lakes. Agents have some mental representation of their environment and know the relative locations of habitat features such as good escape terrain. They are characterized only by their current and previous locations on the raster environment stored as the *x*- and *y*-coordinate of the center of the grid cell. Bighorn agents are capable of perceiving all attributes of the cell they currently occupy, the relative location of escape terrain, along with all parameters in the eight grid cells immediately surrounding their current location. Movement is not goal orientated; instead, a simple rule-based decision-making heuristic guides bighorn movement in this model (Fig. 2).

Bighorn sheep movement is implemented as a pseudo-biased random walk wherein agents will evaluate the quality of habitat immediately surrounding their current location and move towards favourable habitat. Each time step, the bighorn sheep agent will move from its current location to one of eight neighboring cells. Agent movement is probabilistic and driven by the type of land cover found at each cell. First a randomized list of all cells adjacent to the bighorn’s current cell is generated. A cell is selected from that list and evaluated according to the movement rules summarized in Table 1. If the cell is located on a river, lake, or road, or has greater than 40% crown cover, it is removed as a possible cell to move to. If the cell is not on a river, lake or road and has less than 40% crown cover, the cell’s distance to escape terrain is evaluated. If the cell is within 400 m of suitable escape terrain (cells with slope greater than 40 degrees), the agent will move to that cell. If the cell is further than 400 m away, the cell’s distance to escape terrain is evaluated against an equation that relates the probability of movement to distance from escape terrain (Eq. (1)). This is done by drawing a random number between 1 and 100 and if the random number is smaller than the *probability of movement* (Eq. (1)), the agent will move to that cell. Thus, as distance to escape terrain increases, it becomes less likely that a sheep agent will move to that cell. This equation was based off a habitat suitability model constructed for bighorn sheep in the Okanagan Valley (Warman et al., 1998). Note that because each grid cell is 75 m in length and width, the probability of movement effectively decreases beyond 450 m since it is not possible to be exactly 400 m from escape terrain (400 is not divisible by 75).

(1) \begin{eqnarray*}\mathit{probability~ of ~ movement}=188.21{e}^{-0.0016(\mathit{distance})}\end{eqnarray*}

Each cell is iteratively evaluated against the movement requirements summarized in Table 1. As soon as a suitable cell is found, the sheep agent will move to that cell. The list of neighbouring cells was randomized prior to evaluating any cells. If no cell satisfies the movement rules, the agent is removed from the simulation. An agent cannot move backwards (i.e., into the cell it occupied the previous time step) to prevent agents from becoming “stuck” between small numbers of cells.

### Running the model and management scenarios

A series of simulations were created to evaluate each of three research objectives. Model implementation for each simulation is described below. In all cases, the simulation terminates when all agents have been removed from the simulation. Agents are removed when they become “stuck” (surrounded by only poor habitat), leave the spatial extent, or the simulation ends after 2,000 time steps. Every time a sheep agent moves, its current location is stored as an *x*- and *y*-coordinate in a list. After the model terminates, the number of times a bighorn agent used each pixel on the landscape is aggregated into a single map, which is outputted into ArcMap 10.1.

#### Scenario 1: dispersal between subpopulations without management intervention

The purpose of this scenario is to explore how bighorn sheep might disperse from their known locations on the landscape and to assess the degree of connectivity between existing subpopulations. 100 agents were created and placed on a pixel containing known bighorn sheep occurrences from empirical sighting data. The bighorn sheep agents then disperse from their initial locations by following the movement algorithm (Fig. 2). After a simulation ends, the model will re-initiate at a new bighorn sheep location. This is repeated iteratively through all sheep occurrence locations recorded for the real landscape.

#### Scenario 2: potential bighorn connectivity across the landscape

The purpose of this scenario is to explore how bighorn sheep might make broad use of their landscape to facilitate range expansions or range shifts. One bighorn sheep agent is created and placed on the landscape. Instead of starting the simulation from pre-determined locations, its initial location is determined by selecting a random pixel that satisfies bighorn sheep movement requirements (Table 1). The agent traverses the landscape following behaviour rules (Fig. 2). After a simulation ends, the model re-initiates at a new pixel on the environment. This repeats for 20,000 model iterations.

#### Scenario 3: increasing connectivity by restoring a natural fire regime

Historically, frequent and naturally occurring wildfires maintained high-visibility, open forest habitats for bighorn sheep throughout the Okanagan Valley. However, considerable fire suppression in this region has resulted in widespread loss of suitable bighorn habitat with a concurrent loss in connectivity. Fire has been shown to improve and expand bighorn habitat in other regions (Smith, Hardin & Flinders, 1999; DeCesare & Pletscher, 2006). The purpose of this scenario was to explore if interventions like prescribed burns, removing trees, or allowing natural fires to burn, could increase connectivity between subpopulations and into currently unoccupied bighorn habitat. One bighorn sheep agent is created and placed on the landscape. Its initial location was determined by selecting a random pixel that satisfies bighorn sheep movement requirements (Table 1); however, crown cover was removed as a variable bighorn sheep select against. The agent traverses the landscape following behaviour rules (Fig. 2). After a simulation ends, the model re-initiates at a new pixel on the environment. This repeats for 20,000 model iterations.

#### Scenario 4: increasing connectivity through major road interventions

This scenario aimed to identify where roadway interventions such as removing fencing around highways and constructing animal crossing structures over major roads could increase landscape connectivity for bighorn sheep. We increased the spatial resolution to 25 m by 25 m for this analysis. This was done to provide fine enough detail to determine where sheep highway crossing points were. To identify road crossing points, roads were removed from the model to simulate where bighorn sheep would likely travel if roads were not a barrier to movement. One bighorn sheep agent was created and placed on the landscape. Its initial location was determined by selecting a random pixel that satisfies bighorn sheep movement requirements (Table 1). The agent then traverses the landscape following behaviour rules (Fig. 2). After a simulation ends, the model re-initiates a sheep agent at a new pixel on the environment. This repeats for 20,000 model iterations.

### Model validation and sensitivity analysis

Local bighorn sheep experts verified that the movement rules and preliminary corridor maps were realistic and consistent with where bighorn sheep are anticipated to disperse on this landscape (Dyer, BC FLNRO, pers. comm., 2014), thus providing an expert validation of the landscape level results. The model results were subsequently validated against known bighorn sheep occurrence points to ensure that predicted movement was consistent with known sheep locations. No sheep movement or genetic data were available to validate individual-level movement patterns. We therefore tested the sensitivity of the model to assumptions used in the movement rules and ran an extensive parameter search to show how uncertainty in model rules influences results (see Data S1). The selected parameter values used for the simulations described in this paper are those that gave rise to landscape level results that encompassed more than 80% of bighorn sheep occurrence points. A more detailed description of model validation and sensitivity analysis is provided in the Supplemental Information.

## Results

**Figure 3:**
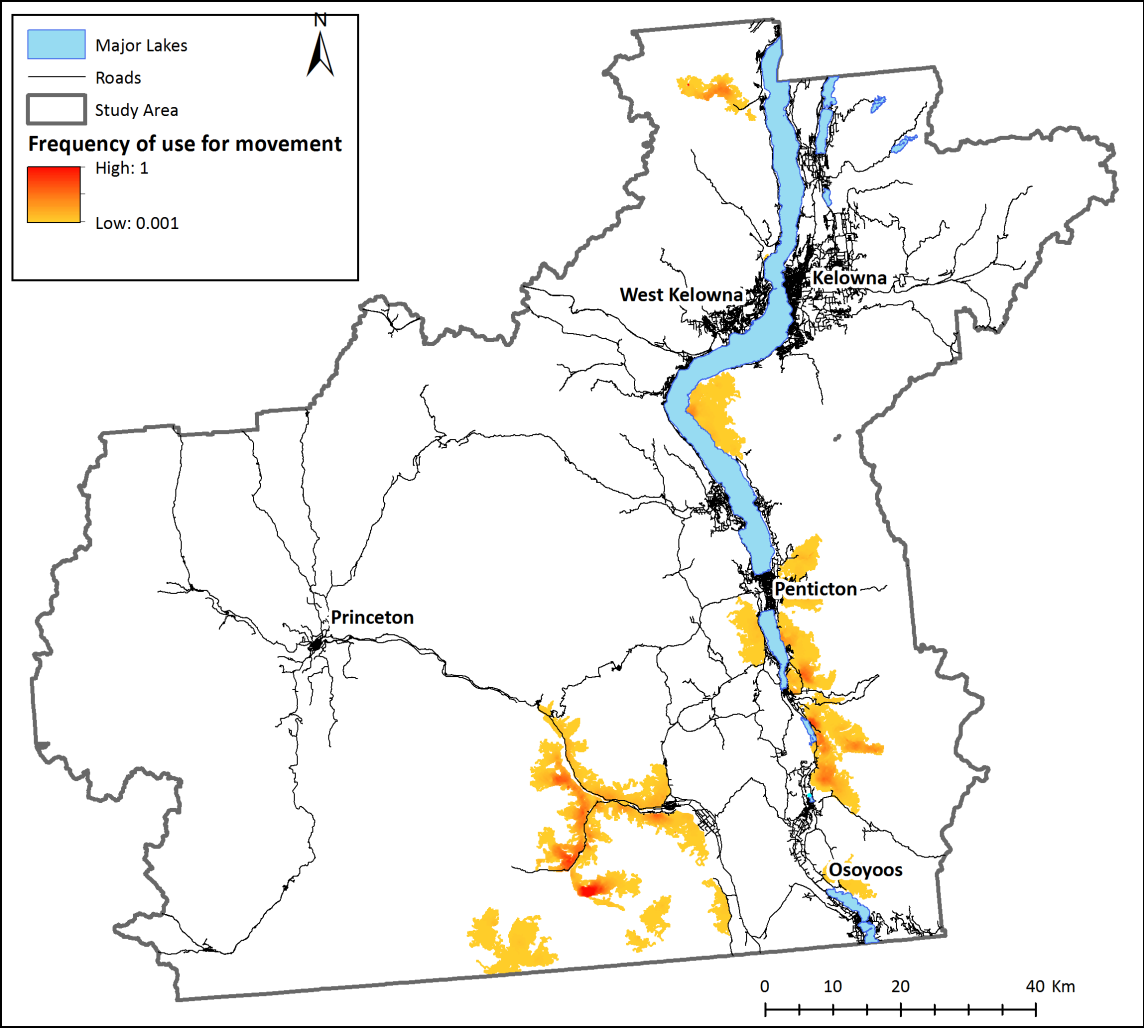
Landscape connectivity for existing bighorn sheep subpopulations. Modeled present-day frequency of use for movement by bighorn sheep, based on simulations with agents starting at known locations of recorded sheep occurrences. 100 agents were placed on the simulated environment at each known bighorn occurrence and allowed to move according to behaviour rules. Relative frequency of use for movement is the number of times each pixel was used for movement by sheep agents divided by the number of times the most used pixel was crossed. White areas are pixels that were never used by a dispersing sheep.

**Figure 4:**
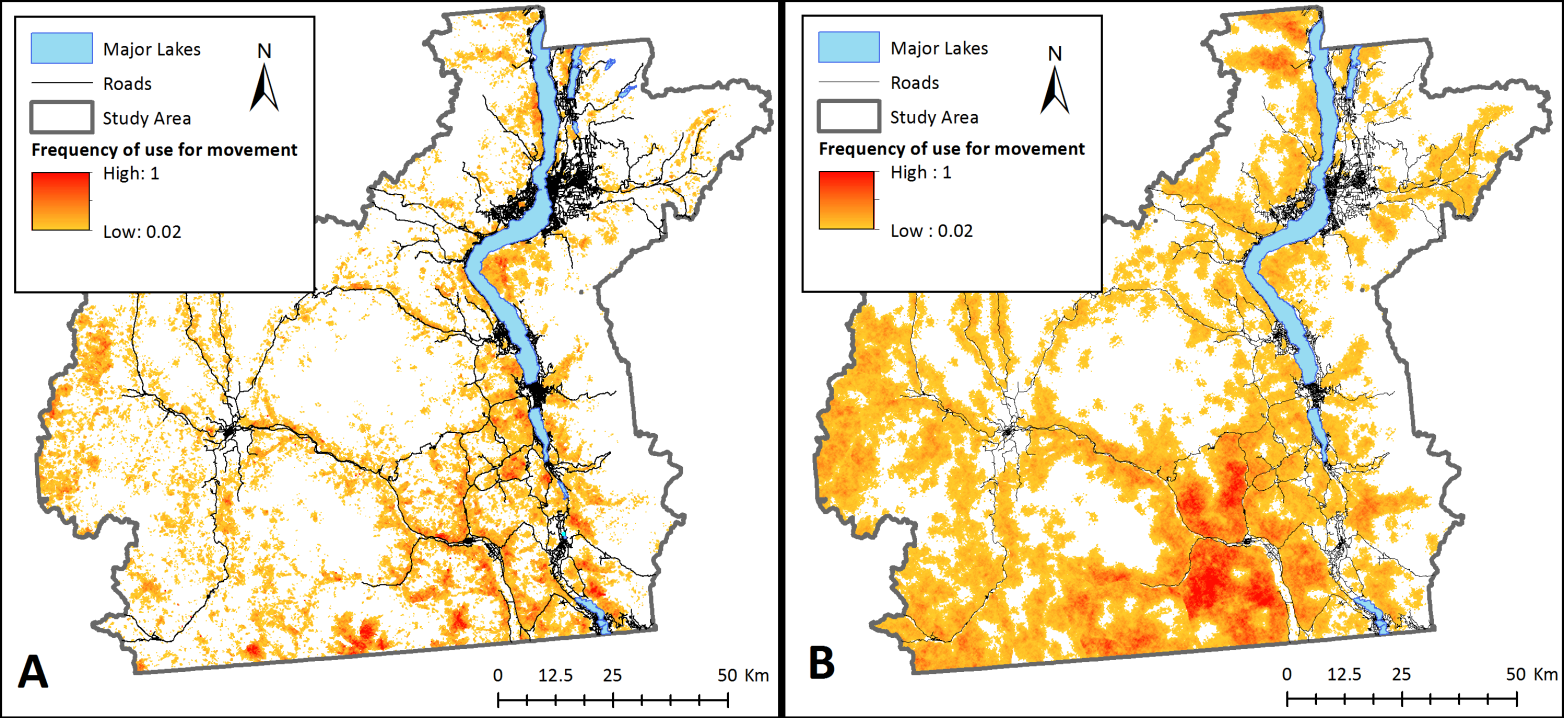
Potential landscape connectivity for bighorn sheep. Potential relative frequency of use for movement by bighorn sheep identified by starting agents at any pixel with suitable habitat for (A) the present-day landscape, and (B) a landscape that simulates management actions that restore natural fire regimes to reduce crown cover. To simulate fire, crown cover was removed as a constraining factor on bighorn sheep movement. Relative frequency of use for movement represents the number of times each pixel was used for movement by sheep agents divided by the number of times the most used pixel was crossed based on 20,000 simulations. White areas are pixels that were never used by a dispersing sheep across simulations.

Based on these modelling results, although areas of good habitat are reasonably well connected within each subpopulation, bighorn sheep agents were unable to reach other subpopulations by dispersing through secure habitat, thus confirming the isolation of existing herds in the study area (scenario 1, Fig. 3). When the individual-based model was modified to start from any pixel that met movement requirements (scenario 2), a mosaic of corridors and stepping-stones are discernable across the landscape (Fig. 4A). Visually, the most used areas on the map appear to follow road transportation corridors.

**Figure 5:**
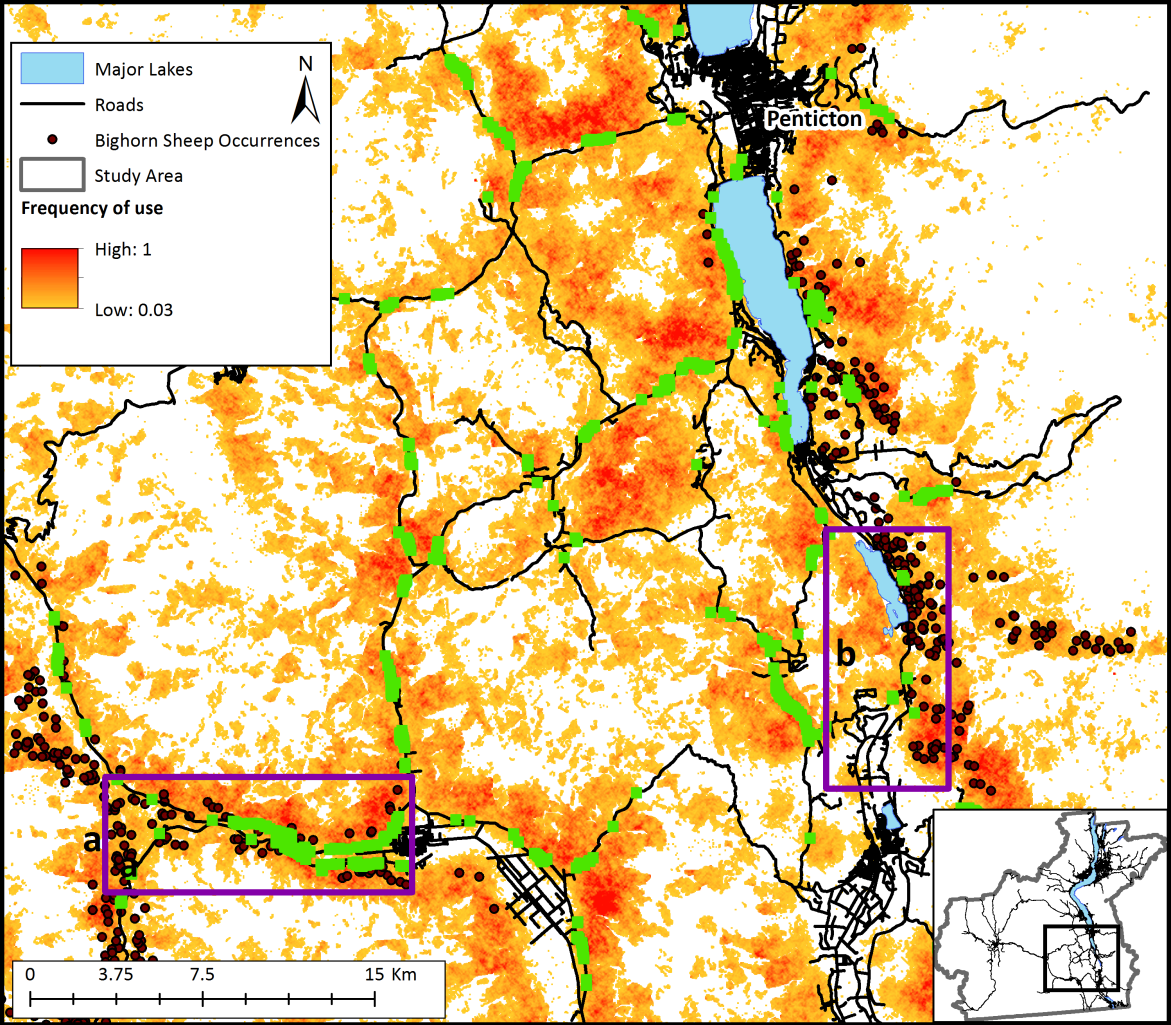
Relative frequency of use of the landscape for bighorn sheep movement and most frequently used road-crossing sites predicted by the bighorn movement model for a section of the study area. Relative frequency of use for movement represents the number of times each pixel was used for movement by sheep agents divided by the number of times the most used pixel was crossed based on 20,000 simulations. White areas are pixels that were never used by a dispersing sheep across simulations. Sections of roads highlighted in green indicate the top 10% most used locations for road crossings by simulated bighorn sheep. The dark purple rectangles (a and b) show regions of proposed highway improvements to facilitate sheep crossings based on model results.

Management interventions that reduce crown cover such as prescribed burns with timber harvest and/or allowing naturally occurring wildfires to burn in bighorn habitat could considerably increase bighorn sheep landscape connectivity in the Okanagan (scenario 3; Fig. 4B). This increase in connectivity is most pronounced in the South Okanagan where the landscape becomes a nearly continuous swath of pixels that were used by simulated sheep. The effect of increasing road permeability was less pronounced (scenario 4; Fig. 5). The top 10% most used road crossing sites were identified in ArcMap (ESRI Inc.) and highlighted (Fig. 5). Additionally, two areas of interest are indicated on Fig. 5. First, a major road that bisects the South Okanagan subpopulation has a nearly continuous range of crossing points across a stretch of several kilometers (indicated with purple rectangle (a); Fig. 5). Second, a road that runs parallel to and between the major lakes and that isolates the Eastern sheep subpopulations was consistently crossed at one point (indicated with a purple rectangle (b); Fig. 5). These identified crossing locations may be the object of road mitigation measures to improve bighorn connectivity in the study area.

## Discussion

Maintaining landscape connectivity has become of central importance to the conservation of many species, especially in fragmented landscapes. Conservation and restoration of landscape corridors and stepping-stones are common attempts to facilitate animal movement across a landscape. However, a dichotomy between methods used to identify corridors and the spatial scale of management efforts somewhat hinders our ability to predict where corridors should be implemented (Chetkiewicz, St. Clair & Boyce, 2006). Our study offers insight into the potential use of individual-based models to evaluate landscape connectivity by predicting where corridors and stepping-stones are located on a landscape. Here we have shown how a relatively simple individual-based model with behaviourally realistic movement rules can estimate connectivity across a landscape.

The results from our model suggest there are no existing continuous connections between sub-populations of bighorn sheep (Fig. 3). Although no formal genetic work has been completed for sheep in this region, our findings support anecdotal observations made by site- and species-experts that there has been little to no exchange of individuals between subpopulations in recent years (Dyer & Reid, BC FLNRO, pers. comm., 2014). Despite this, our results indicate that the Okanagan landscape has potential to facilitate bighorn movement into currently unoccupied, yet suitable, bighorn habitats (Fig. 4A). This is important given the anticipated re-structuring of habitat as a result of changing climates and increasing pressure on natural habitats due to human land use in the region (Nuñez et al., 2013). Many of the most used areas in Fig. 4A follow transportation corridors. We believe this result was observed because roads in this region follow the natural contours of the landscape and are thus adjacent to steep and rocky regions. By highlighting regions of high connectivity in close proximity to roads, this result re-inforces the importance of evaluating how roads can be made more permeable to sheep without increasing collision risk. Fig. 4 also identifies stepping-stone connections between subpopulations that could provide connectivity in areas where continuous corridors were not identified in Fig. 3. Interestingly, site experts who are familiar with historic bighorn sheep ranges in the Okanagan Valley indicated the results in Fig. 4A highlight regions of the landscape that are known to have previously supported bighorn sheep populations (Dyer & Reid, BC FLNRO, pers. comm., 2014).

The results of scenarios that could increase bighorn sheep connectivity demonstrate that a combination of road interventions along with restoring natural fire regimes in key areas of the study area are likely to increase bighorn connectivity. There is currently interest from various levels of government to install a wildlife bridge across a major road that bisects the Southern bighorn sheep population. Our model suggests that bighorn sheep are likely to use such a crossing structure at any point along that road (Fig. 5; purple rectangle (a)). This is an important finding which provides for flexibility to the road crossing implementation process, allowing a potential structure to be located where land ownership and tenure are most amenable. In addition to road interventions, there appears to be considerable opportunity to restore dispersal ability between subpopulations by removing crown cover (either through prescribed burns or tree removal). It is, of course, unrealistic to remove crown cover across the entire landscape as we simulated in the model. However, the results of this exercise show how fire or management interventions that mimic fire could positively affect bighorn sheep dispersal on this landscape. Bighorn sheep occupy a wide range in western North America extending from British Columbia, Canada to California, USA (Demarchi, Hartwig & Demarchi, 2000). The population of sheep we focused on in this study occupy the most northern tip of the greater bighorn range. Maintaining connectivity of these northern habitats is important on a continental scale to promote species’ future range shifts in a changing climate. While this model has focused on subpopulations found in southern BC, it is easily transferable to other regions, and could be run for other landscapes with a simple adaptation of the input files describing landscape topography and vegetation cover.

We have identified several key advantages of an IBM modeling approach to inform land use management practices that aim to increase landscape connectivity. First, this approach allows researchers to explore if physical linkages exist on the landscape, and if not, where the major connectivity gaps are. Although it is possible, and probably more accurate, to use genetic indices to determine whether connectivity exists between subpopulations (Manel et al., 2003; Hepenstrick et al., 2012), many conservation efforts face a paucity of resources that limits the accessibility of genetic analyses. Least cost path has been applied to assess connectivity gaps by incorporating a maximum cumulative cost distance (for example, Epps et al., 2007). However, application of LCP without considering the accumulated-cost of a least cost path may result in routes that do not capture sufficient information about the ecological costs of movement across a resistance layer (Etherington & Holland, 2013). While it may be useful to identify ideal corridors through poor habitat that could be made more useable with restoration work, least cost path risks overestimating connectivity between sub-populations by showing routes through poor habitat that may be unlikely to facilitate movement. Circuit theory on the other hand, better simulates landscapes from the perspective of an individual and has proven useful for exploring edge-crossing tendencies (St Louis et al., 2014). An IBM, such as the one we present here, could provide a reasonable indication of whether there is existing connectivity between subpopulations when a sufficient understanding of animal movement behaviour is available to validate and parameterize model assumptions. Individual-based models of animal flow across a landscape often incorporate a correlated random walk or consider trade-offs related to energy or fitness to mimic how an animal might move across its landscape (Aben et al., 2014). A random walk suffices in the following since this analysis is not looking at specific trajectories of sheep but rather the frequency of selection of landscape cells over many model iterations.

The second major advantage we identified with an IBM over the more traditional least cost path approach is that an IBM simulates individuals as boundedly rational (i.e., responding to local information cues without having perfect knowledge of the whole system). It is widely acknowledged that connectivity is a process that emerges as the result of how a species perceives and reacts to its landscape along with the structural composition and configuration of landscape elements (Tischendorf & Fahrig, 2000; Walpole et al., 2012). Good connectivity indices therefore should realistically restrict the perceptual range of simulated individuals. By simulating a random walk, circuit theory also approximates individuals as boundedly rational and identifies corridors based on a limited perception of the landscape. In contrast, an assumption implicit with LCP analysis is that animals are capable of perceiving their entire landscape and identifying optimal routes between pre-determined locations. Although it is reasonable to assume animals have a general understanding of their landscape including the locations of good habitat, it is unlikely an individual is capable of discerning the most optimal route between these locations. In our approach, agent movement was informed by local landscape variables. By realistically limiting the amount of information agents use to make movement decisions, an IBM may capture non-optimal dispersal behaviours ubiquitous in natural systems.

The third advantage of an IBM is that we can move away from the patch-corridor archetype to conservation towards a more nuanced representation of habitat quality on a landscape. Traditional approaches to connectivity analyses distinguish between core areas and corridors. Conversely, the IBM presented in this study highlights how a species might make broad use of a landscape for dispersal or migration without differentiating between habitat areas and corridors. It is important to note this type of analysis cannot replace a habitat suitability model, which considers ecological requirements of species, climate envelopes, and limiting factors to predict the likelihood of species occurrence across a landscape (Hirzel & Le Lay, 2008). However, this model provides additional insight into how a species might use a landscape over deterministic, single-best-route connectivity analyses such as least cost path (but see Cushman, McKelvey & Schwartz, 2009). Although habitat corridors are often considered to be a panacea for fragmented habitats, other features of a landscape such as stepping-stones have the propensity to facilitate dispersal between larger habitat patches. The individual-based model presented here identified such features of a landscape in addition to corridors.

Additional work remains to be done to improve the utility of individual-based models for assessing landscape-scale connectivity. It is important to emphasize that the results presented here show likely corridors for bighorn sheep use across a landscape; however, it is difficult to claim these results show functional connections without extensive bighorn sheep movement data. By only simulating dispersal through secure habitat, we have effectively assumed that structural connectivity is synonymous with functional connectivity. For individuals in fragmented landscapes, dispersal through insecure habitat is well recognized as a central process contributing to functional connectivity (Baguette et al., 2013). The type of habitat a species is moving through heavily influences dispersal strategies. For example, an individual attempting to cross a large area of poor habitat is likely to move quickly with a high degree of correlation in the direction of successive movements. This is in contrast to the same individual travelling through good habitat, which might result in a more circuitous and slow route. In this study, the decision to restrict simulated movement to areas of good habitat was made for two reasons. First, and most importantly, a conservation priority on this landscape is to identify, maintain and/or restore physical linkages between areas of good habitat for bighorn sheep. We therefore designed and implemented this model to identify routes that highlight how a sheep might travel through secure habitat towards subpopulations or unoccupied areas. Second, bighorn sheep are extremely cautious animals. Even while dispersing, bighorns are observed to follow rocky ridges and avoid densely forested areas. This prompted our modeling decision to identify routes that highlight how a sheep might travel through secure habitat towards subpopulations or unoccupied areas. An interesting direction for future work is to identify good gap-crossing locations through poor habitat by coupling the IBM described here with a least cost path model. Land use managers could then direct efforts to preserve crossing points or make them less hazardous for sheep.

A second limitation of this study is it captures the average behaviour of a population at the expense of individualized responses to the landscape. Dispersal is a multi-causal process with considerable variability within a population (Kokko & Lopez-Sepulcre, 2006). Individuals likely show a spectrum of behaviours in response to landscape elements; consequently, movement trajectories will differ substantially across a population (Elliot et al., 2014). This model was created by establishing a list of rules that all agents follow with no variation among individuals. Although this shows how the population as a whole might use a landscape, it does not capture individuals that show a stronger tendency to travel across barriers. We have effectively simulated the “worst-case” connectivity scenario where individuals are incapable of crossing gaps in good habitat, and thus, this model likely underestimates connectivity. In the real system, individuals may undertake rare dispersals across more difficult terrain, thus facilitating gene flow, colonizing new habitats, and expanding ranges. A priority for future work is to incorporate inter-individual variability into model assumptions. Individual-based models are an excellent platform to simulate disparate responses to landscape elements. However, to incorporate this into the model described here, we would require an extensive understanding of the variation in behaviours found in bighorn sheep, which is relatively unexplored for the study area.

An important task in projects that aim to restore landscape connectivity is linking connectivity into more general conservation plans, which often have a spectrum of objectives. The connectivity approach outlined here uniquely highlights all components of a landscape that contribute to connectivity, not just the most optimal corridors or paths of least resistance. On-the-ground conservation work tends to be a balancing act between where and when opportunities for conservation present themselves alongside where efforts are most needed. Land use managers could use this methodology to identify where corridors “fit” with other conservation priorities for a particular landscape. Moreover, most conservation projects tend to have limited resources to restore and improve connectivity. We have shown how an IBM can be manipulated to explore the impact of management scenarios on landscape connectivity before investing in expensive on-the-ground work. With rapid and considerable habitat fragmentation as a result of human land-use and climate changes, it is critical that decision support tools guide conservation work in the most effective direction to meet the needs of species.

## Conclusions

Maintaining functionally connected landscapes is aptly important for conservation, particularly for facilitating range shifts in response to climate change (Krosby et al., 2010). While conservation and restoration of habitat to produce simple linear corridors may be effective strategies for facilitating species movement through hostile environments, achieving effective connectivity and viable animal populations at the landscape scale will require more than corridors. The use of an IBM in this study highlights the power of this method to identify how a species might make broad use of a landscape for movement and migration. It identifies connectivity to areas that are suitable yet not currently occupied emphasizing the importance of designing conservation plans that encompass more than just current species ranges. The individual-based approach also provided a more realistic representation of how animals perceive and move in their habitats than traditional approaches to identify corridors such as least cost path analysis. We would recommend further application of this approach in connectivity studies, particularly for species where facilitating range expansions is a priority.

## Supplemental Information

10.7717/peerj.2001/supp-1 Data S1 Description of the model using the Objectives, Design concepts, and Details (ODD) protocol

10.7717/peerj.2001/supp-2 Table S1 Source data layers used to create and parameterize models This table lists the geospatial data layers used as input to the model, and the sources of these data.

10.7717/peerj.2001/supp-3 Supplemental Information 1 Individual-based bighorn sheep movement model Original source code files for the bighorn sheep movement model. Files are written in Java for the Repast Symphony modelling environment.

## Additional Information and Declarations

### Data Availability

The following information was supplied regarding data availability: The model code, written using the Repast Simphony agent-based modelling toolkit, is provided as Data S1.
