# Supplementary material for: An individual-based modelling approach to estimate landscape connectivity for bighorn sheep (Ovis canadensis)
Source: PeerJ. 2016 May 5;4:e2001. doi: 10.7717/peerj.2001 (PMC4860333; doi:10.7717/peerj.2001)
Supplement: Data S1 [file peerj-04-2001-s001.docx]

**Supplemental data S1: IBM Objectives, Design concepts, and Details (ODD) protocol**

## 1. Purpose

The Individual based model presented here is designed to simulate individuals wandering across a spatially explicit landscape using simple and species-specific movement rules. The overarching purpose of this model is to identify habitat corridors on a landscape by aggregating the movement paths of individuals across multiple model iterations. This model was also designed to test the effectiveness of various management scenarios that aim to increase landscape connectivity.

## 2. Entities, state variables, and scales

This model is composed of three entities: a landscape of discrete grid cells, bighorn sheep agents and a temporal scale. These are described below.

*The landscape*- the landscape is conceptualized as a series of heterogeneous grid cells 75 m x 75 m in size. Each grid cell has the following attributes: a spatial coordinate, slope (in degrees), percent crown cover, and the presence or absence of lakes, rivers, and roads. The landscape is static and shows no diurnal or seasonal variation.

*Bighorn sheep agents* – bighorn agents are characterized only by their current location and previous location within the environment. Locations are stored as the x- and y-coordinate of the center of the grid cell. Bighorn agents are capable of perceiving all attributes of the grid cell it currently occupies along with the eight grid cells immediately surrounding its current location. Furthermore, bighorn agents are assumed to have some knowledge of their surroundings and therefore know relative distances to suitable escape terrain. Movement is not goal orientated; instead, a simple rule-based decision-making heuristic guides bighorn movement in this model.

*Temporal scale* –A series of discrete “ticks” is used to track the current state of the model. Each tick has no real-world value.

## 3. Process overview and scheduling

The model begins when a bighorn sheep agent is placed on a landscape. The initial location of a bighorn sheep agent is randomized; however, an agent cannot start in poor habitat. After initialization, the model proceeds by iterating through the *move* sub-model until the agent either becomes “stuck” (no valid movement option exists), leaves the spatial extent, or after 2000 time steps. At this point, the simulation terminates and the spatial coordinated of every cell on the landscape used during the bighorn’s route is recorded. Implementation details of the *move* sub-model are described in detail below.

## 4. Design concepts

*Basic principles.* This model was designed to capture critical processes underlying bighorn sheep movement behavior within the Okanagan Valley, British Columbia, Canada as a means of identifying habitat corridors. Bighorn sheep movement is implemented as a pseudo-biased random walk wherein agents will evaluate the quality of habitat immediately surrounding their current location and move towards favourable habitat. The bighorn agent will continue moving across the landscape until either becoming “stuck” or leaving the spatial extent. To identify habitat corridors, the movement paths used by agents across multiple model iterations are overlaid into a single file.

*Emergence*. Habitat corridors emerge as a result of overlaying the individual movement paths used by agents across multiple model iterations.

*Adaptation*. Bighorn sheep agents will adapt by moving towards habitat cells of good quality.

*Objectives*. Agents in this model have no objective. Movement is not goal orientated.

*Sensing*. Bighorn sheep agents are able to perceive the habitat found at their current location and in the eight grid cells immediately surrounding their current location. Moreover, bighorn sheep agents are assumed to have some understanding of their landscape and are therefore able to sense the relative distance to important landscape structures including steep terrain.

*Stochasticity*. Stochastic processes are used to create two sources of variability in this model. First, the initial bighorn sheep location is determined through a stochastic process wherein a random grid cell is chosen and then evaluated to determine if it meets bighorn sheep habitat requirements. If yes, the sheep agent is placed at that location. If no, the habitat at a second randomly selected grid cell is compared to the bighorn sheep habitat requirements. This process is repeated until an appropriate initial grid cell is found. Second, if during the *move* sub-model multiple cells are determined as appropriate to move towards, a random process is used to select a cell to move to out of a list of possible good cells.

*Observation*. Each time step, the x- and y- coordinate of the bighorn sheep is exported to a text file.

## 5. Initialization

To create the environment, an ASCII text file of each landscape layer including roads, rivers, crown cover, lakes, percent crown cover, and slope is imported into the programming environment. A matrix is then created within the programming environment for each landscape layer. The values in each ASCII text file are subsequently read and assigned to their corresponding cell within the grid. After the landscape has been created, a single bighorn sheep agent is placed on a randomly selected grid cell. The sheep agent will then evaluate the habitat at that grid cell. If the cell meets all bighorn habitat requirements, the model will execute. If not, the sheep is re-located to another randomly selected grid cell and the process repeats. This continues until a suitable grid cell is located.

## 6. Input data

An ASCII text file for each landscape layer including roads, rivers, crown cover, lakes, and slope is imported into the programming environment. We created these input files by converting raster maps of each layer into an ASCII text file in ArcGIS 10.1.

## 7. Submodels

*Move submodel*

The movement sub-model defines exactly how bighorn sheep choose a neighbouring cell and subsequently move to that cell. First, the current grid cell is queried and a list of the eight grid cells immediately surrounding the current cell is created. Any cell within that list containing a lake is removed from the list (lakes are an absolute barrier to bighorn sheep movement in this model and therefore are not considered). Each cell within the list is then evaluated against the following criteria. Any cell not meeting one or more criteria is removed from the list of possible movement cells. (1) the cell cannot be the previously occupied cell; (2) the cell must be an appropriate distance to escape terrain; (3) the cell must not have more than 40% crown cover; and, (4) the cell most not be a road or a river. Once a cell has been found that meets all of the criteria, the agent will move to that cell. If no cells meet the criteria, the agent is stuck and the simulation terminates.

If a cell is within 400 m of escape terrain, it satisfies the distance to escape terrain requirement and the agent will move to that cell. If the cell is greater than 400 m from escape terrain, then its distance from escape terrain is evaluated against equation 1, which relates the probability of movement to distance from escape terrain. This is done by drawing a random number between 1 and 100 and comparing the random number to the solution of Eq. 1. If the random number is smaller than the *probability of movement,* the agent will move to that cell. Thus, as distance to escape terrain increases it becomes less likely that a sheep agent will move to that cell.

𝑝𝑟𝑜𝑏𝑎𝑏𝑖𝑙𝑖𝑡𝑦 𝑜𝑓 𝑚𝑜𝑣𝑒𝑚𝑒𝑛𝑡=188.21e^-0.0016(^*^distance)^* Eq. 1

We based the equation for probability of movement beyond 400 m off of a habitat suitability model constructed for bighorn sheep in the Okanagan Valley (Warman et al. 1998). In this study, researchers identified habitat within 400 m of escape terrain as good (a value of 1 on a 5 point scale with 1 being good habitat and 5 being poor habitat) with the likelihood of use decreasing to “greater than 1600 m” (a value of 5 in the habitat suitability model). We made the assumption that use of an area decreases exponentially with distance to escape terrain, and derived the equation used in our model based on the ranking system used in Warman et al. (1998). Note that because we were working with integer grid cells with a length of 75 m, the probability of movement effectively decreases beyond 450 m since it is not possible to be exactly 400 m from escape terrain (400 is not divisible by 75). Figure 1 shows the likelihood of movement for a given distance from escape terrain.

## Figure 1. The probability of movement (%) to a particular grid cell given the distance to escape terrain.

## 8. Model verification, justification, and sensitivity analyses

Model justification and verification

Bighorn sheep have been extensively studied in the Okanagan Valley and greater bighorn range. We therefore had a plethora of published data along with anecdotal observations from bighorn sheep experts at our disposal. Prior to creating the Okanagan Valley Animal Movement Simulator for bighorn sheep, we preformed an extensive literature search drawing on site-specific government reports retrieved from EcoCat, the Ecological Reports Catalogue of BC in addition to peer-reviewed literature.

From this literature search, we established a list of bighorn sheep movement criteria (table 1). Prior to implementing the model, our bighorn movement criteria was verified for accuracy by site-specific, bighorn sheep experts including a representative from BC Parks, two Ecosystems Biologists and a bioterrain expert working for the Ministry of Forests, Lands and Natural Resource Operations in British Columbia, Canada. Using their feedback, the bighorn movement criteria was modified appropriately and implemented into the individual based model. Local, bighorn sheep experts verified preliminary corridor maps were realistic and all feedback given was incorporated into the model. In addition to expert validation, we compared the simulated habitat corridors to bighorn sheep occurrence data in the Okanagan Valley. 82% of bighorn sheep occurrences were within a simulated habitat corridor. Note that we did not strive for 100% of bighorn sheep occurrences to fall within a corridor because it is possible sheep were observed in poor habitat. Moreover, some of the occurrence points documented were from several years ago; it is possible these locations were recently developed. Similarly, we didn’t limit our model to those locations because the vast majority of habitat suitable for bighorn sheep in the Okanagan is currently unoccupied. Moreover, occurrence data is not a direct reflection of habitat corridors. Although there was no bighorn sheep movement data available to compare our model results against, we are confident the corridors in this model are a realistically predict the location of potential bighorn sheep corridors for this region. Table 1 reflects the final version of movement rules as implemented in the model.

Table 1. Bighorn sheep movement characteristics

| *Movement characteristics* | *Description* | *Corresponding rule in the model* | *References* |
| --- | --- | --- | --- |
| Close proximity to escape terrain | Steep terrain with interspersed rocky outcrops. | Escape terrain is defined in the model as any slope greater than 40 degrees. Bighorns attempt to remain within 400 m of escape terrain with the likelihood of a sheep travelling further than 400 m from escape terrain decreasing as described by $y=188.21e^{-0.0016x}$. This equation was derived from a habitat suitability model previously developed for Okanagan bighorn sheep (Warman et al., 1998) | (DeCesare & Pletscher, 2006; Demarchi et al., 2000; Shannon et al., 1975; Smith, Flinders & Winn, 1991; Tilton & Willard, 1982) |
| Preference for sparse vegetation | Bighorn sheep avoid densely vegetated areas or areas with a closed canopy | Sheep will not occupy a cell with more than 40% crown cover | (DeCesare & Pletscher 2006; Demarchi et al., 2000; Smith et al., 1991) |
| Roads | Bighorns sheep are severely deterred by roads, particularly highways and major streets | Sheep cannot occupy a cell with a road | (Epps et al., 2005; MacArthur, Johnston & Geist, 1979) |
| Ability to cross rivers and lakes | Bighorn sheep rarely cross rivers and never cross large water bodies | Sheep cannot occupy a cell with a lake or river | (Demarchi et al., 2000) |

After finalizing model rules, we determined the optimal number of model iterations by varying the number of iterations and comparing the total number of cells visiting on the landscape across simulations. The number of cells visited did not change considerably when the number of model iterations was increased past 20 000.

Sensitivity analysis

Given the uncertainty associated with the amount of crown cover and steepness of slope bighorn sheep responds to, we preformed an extensive sensitivity analysis to explore how these two variables influence habitat corridors. The following simulations were explored:

- slope > 40 with crown cover < (0, 20, 30, 40, 50, and 60)
- slope > 30 with crown cover < (0, 20, 30, 40, 50, and 60)
- slope > 20 with crown cover < (0, 20, 30, 40, 50, and 60)

These sets of parameters were chosen because they are a realistic range over which bighorn sheep *could* select habitat for movement. Results from sensitivity analyses indicate that the overarching pattern of where habitat corridors were identified remains static regardless of the value given to slope and crown cover (Fig 1.). The width and connectedness of these corridors however, varies as we increased or decreased the steepness of escape terrain sheep required by bighorns or amount of crown cover. In the final model, slope greater than 40 degrees with crown cover less than 40% was used.


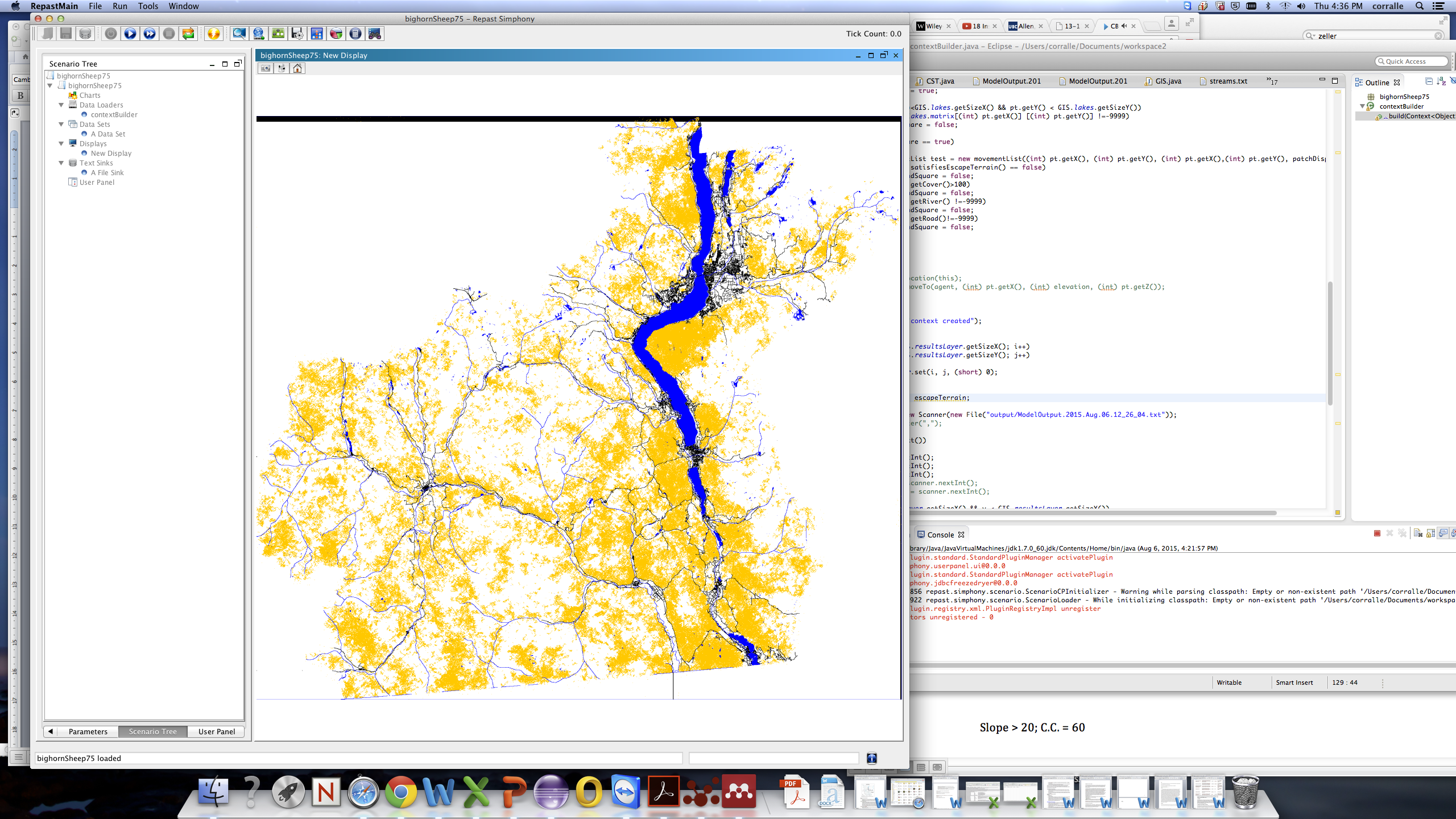

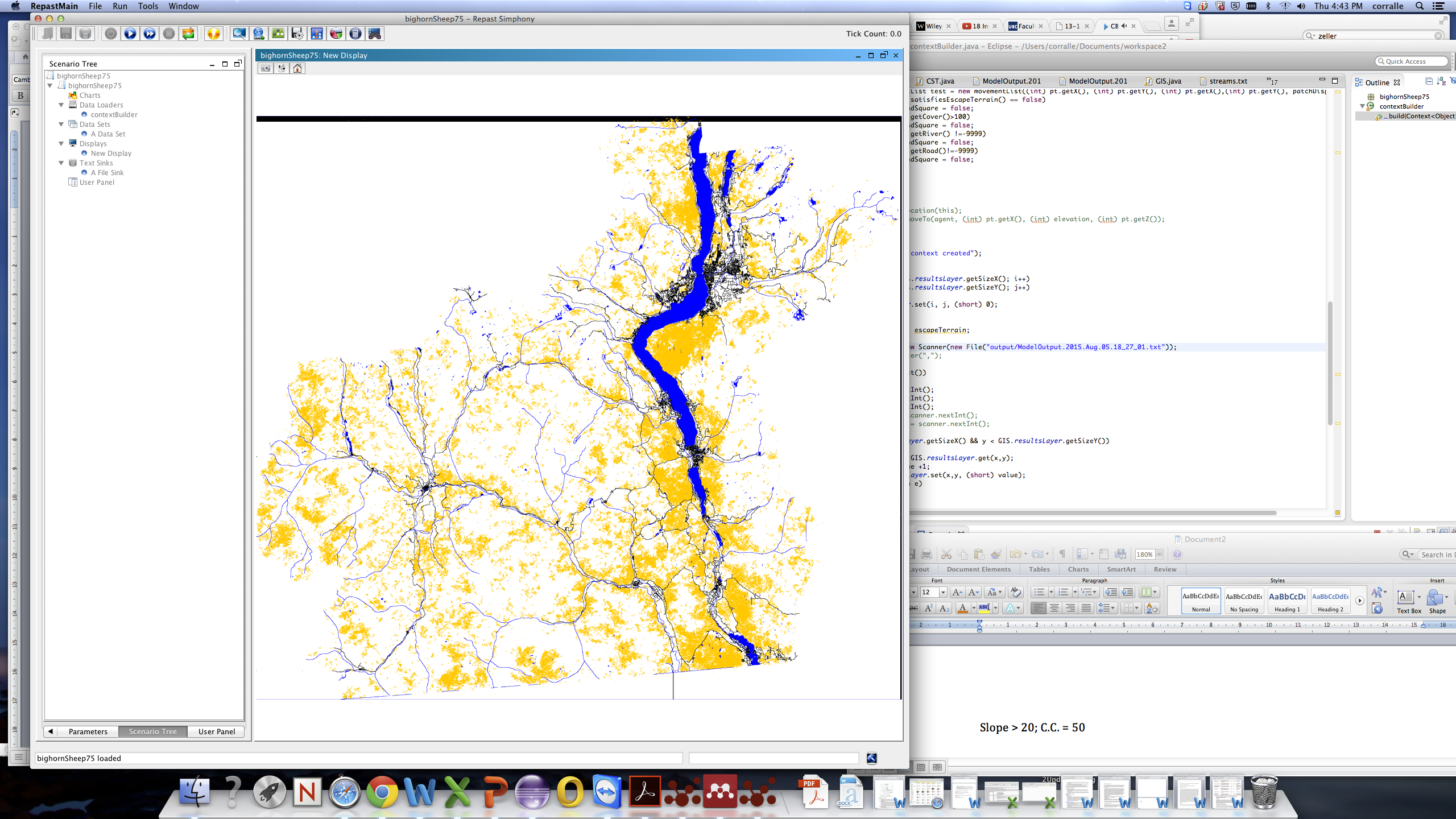

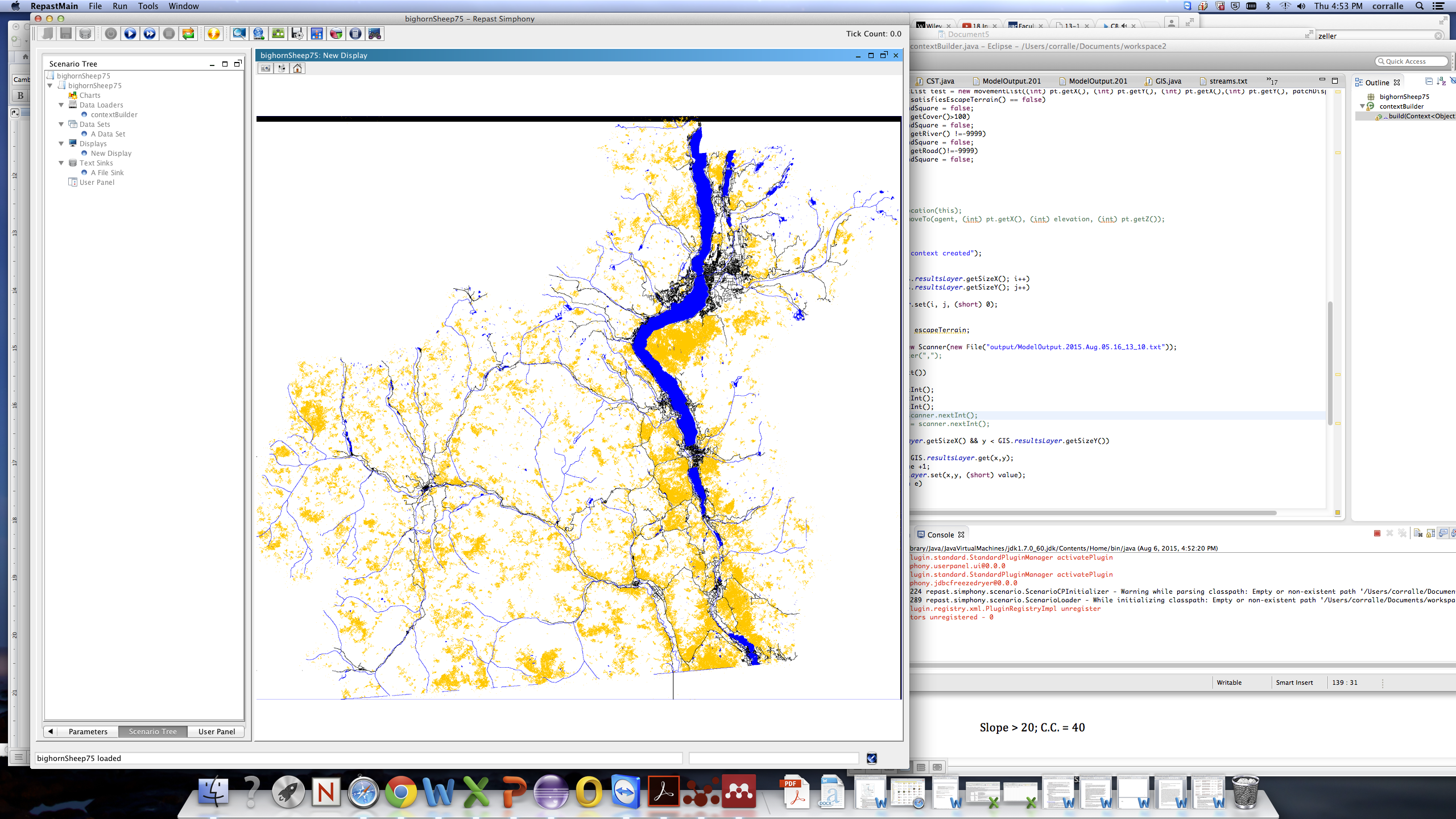

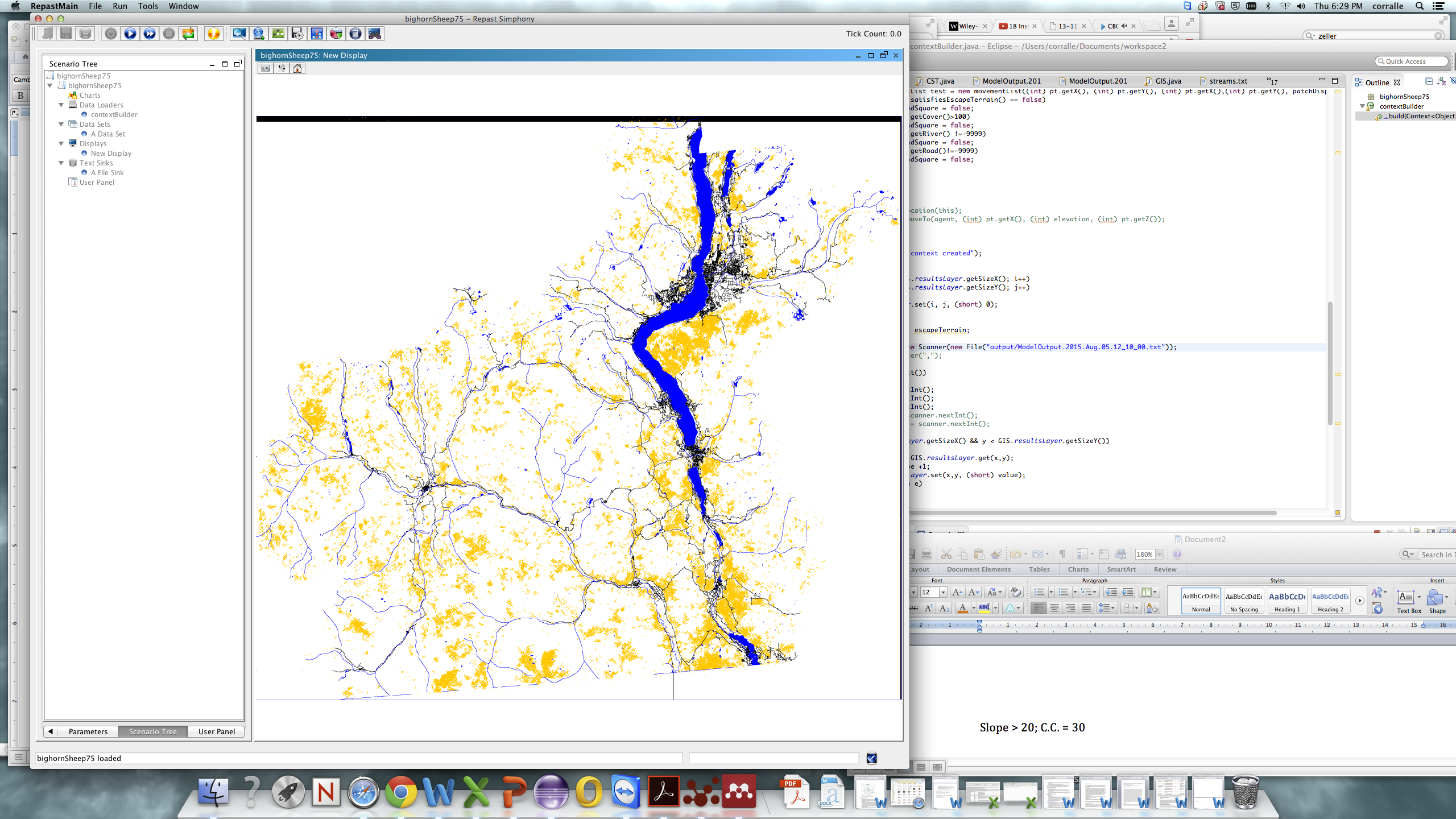

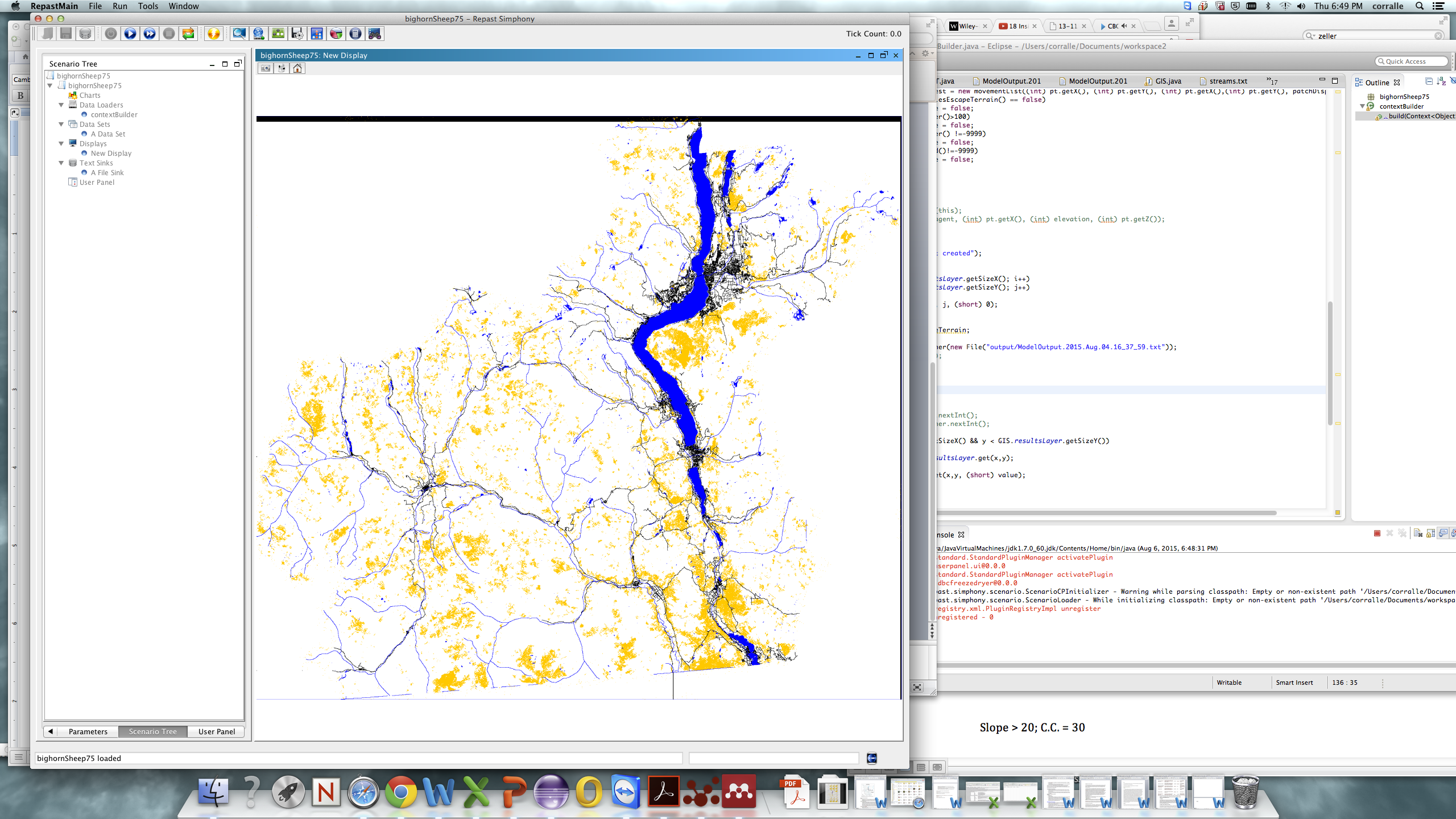

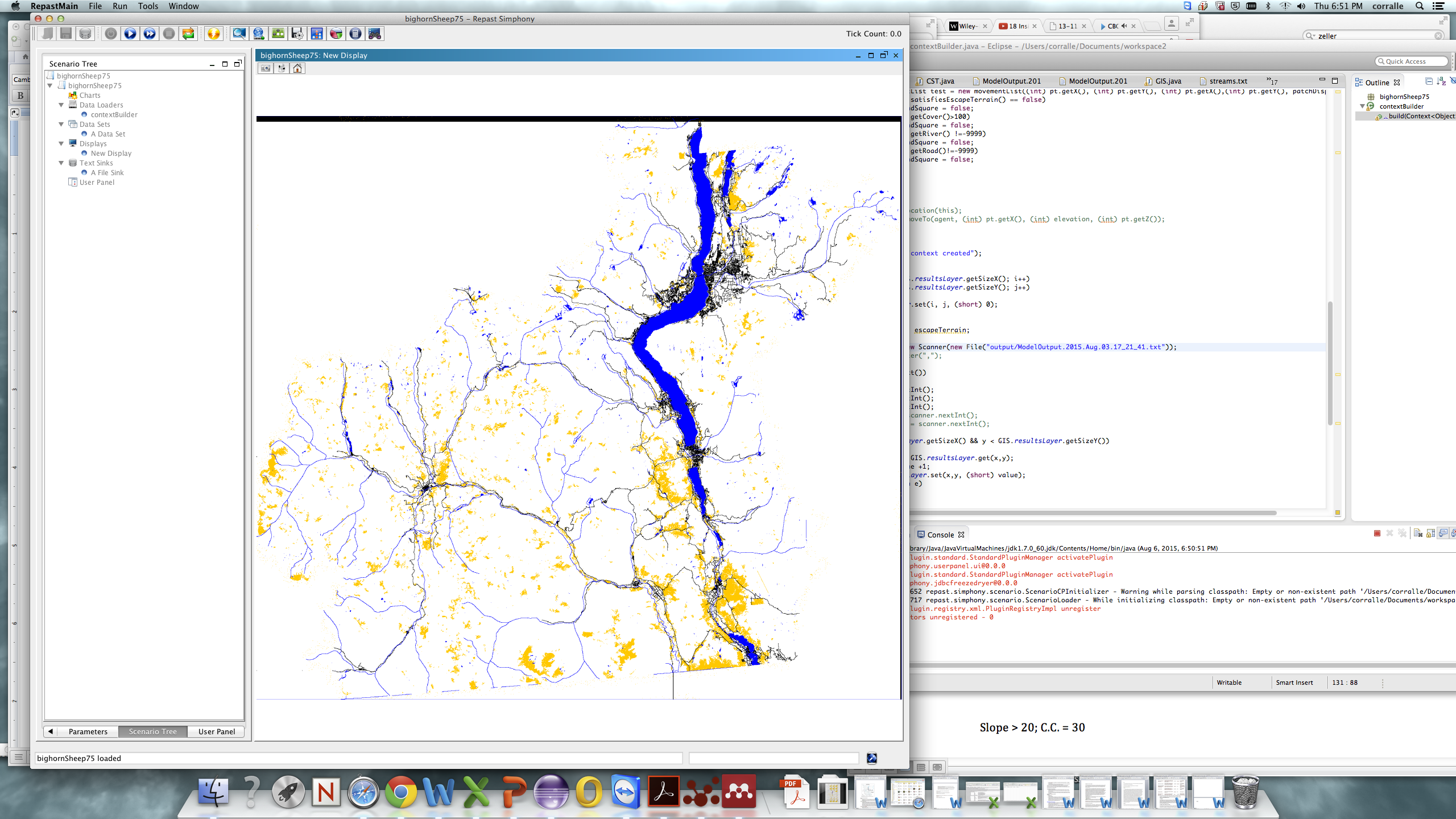

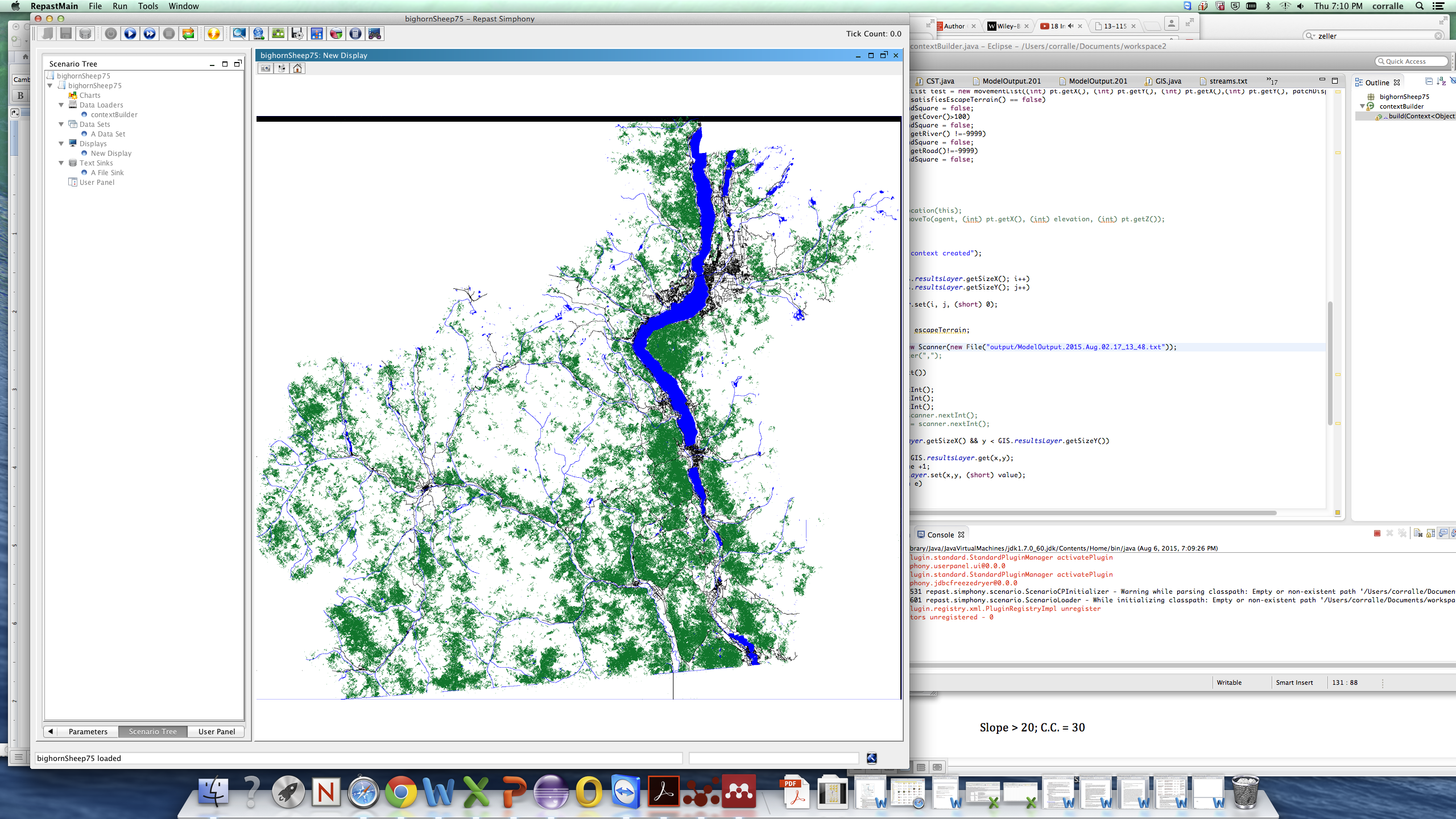

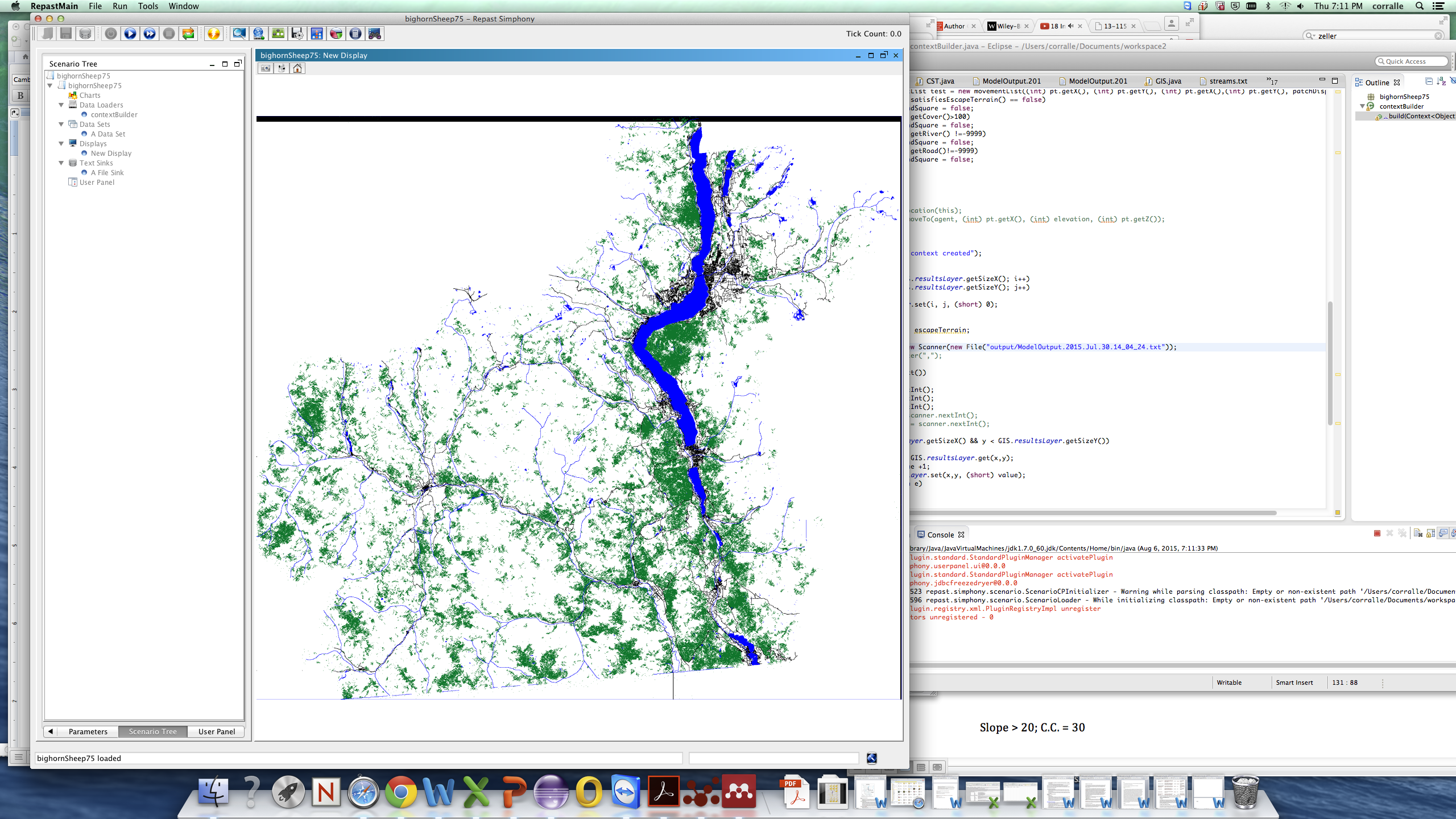

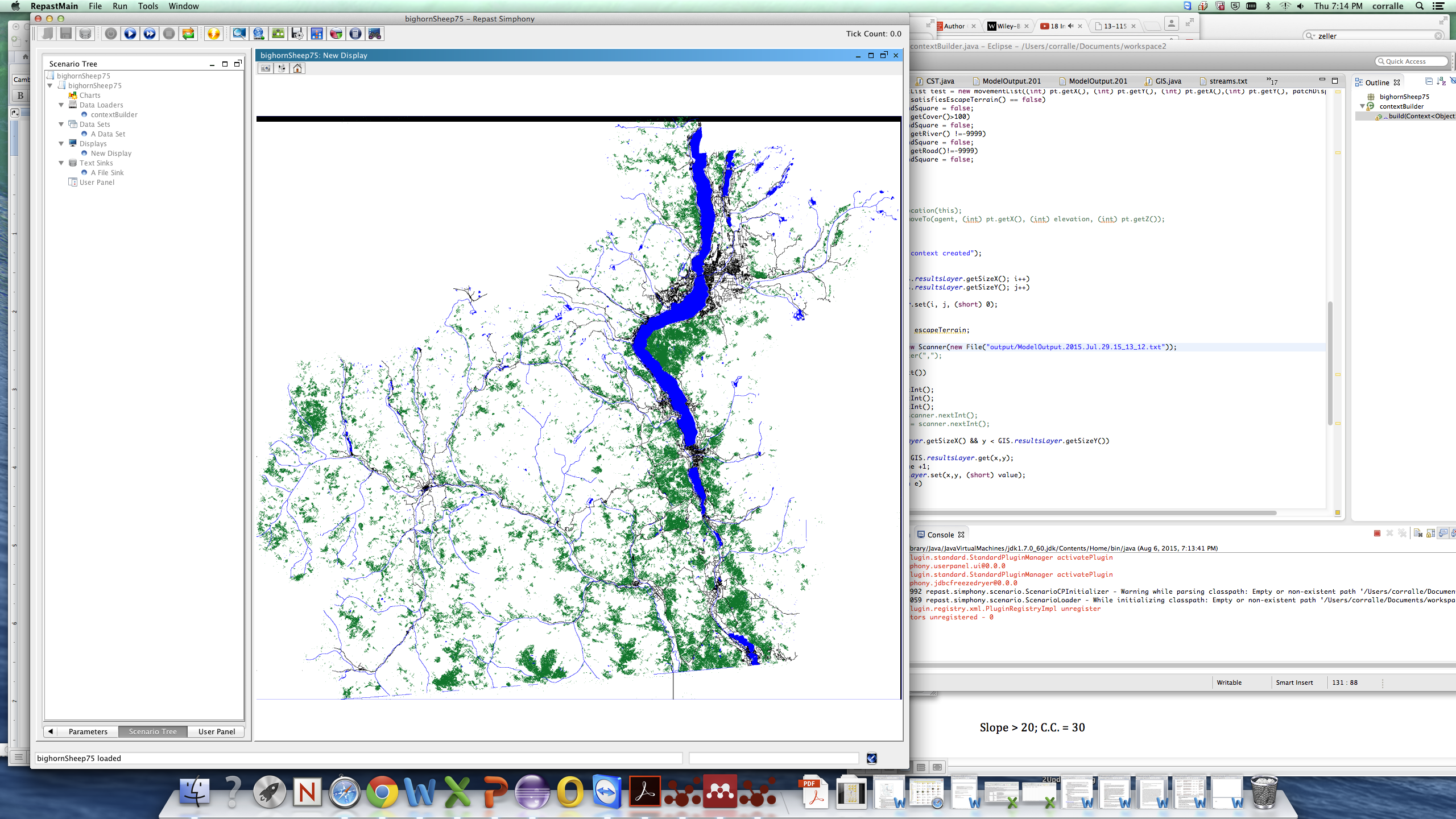

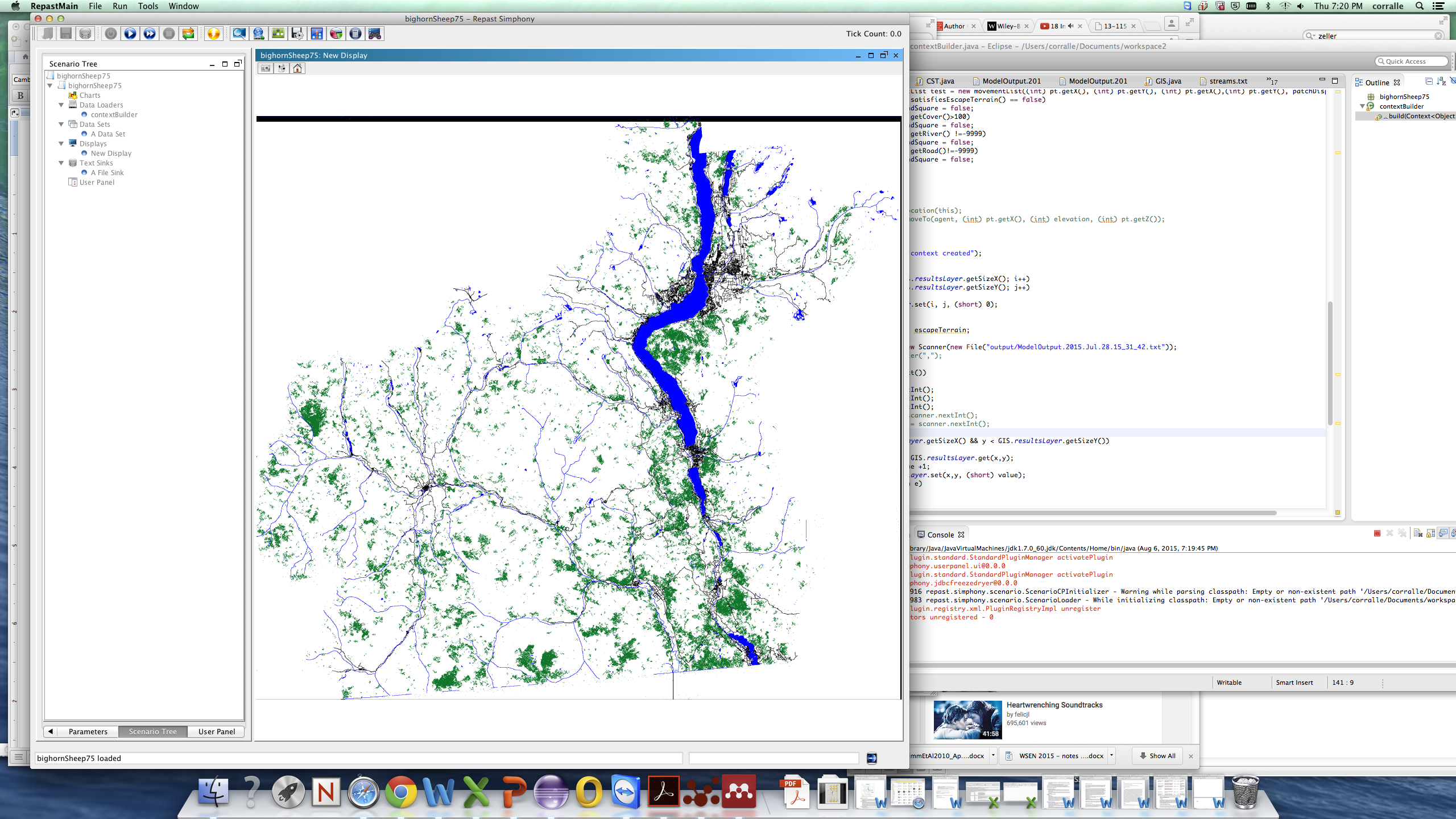

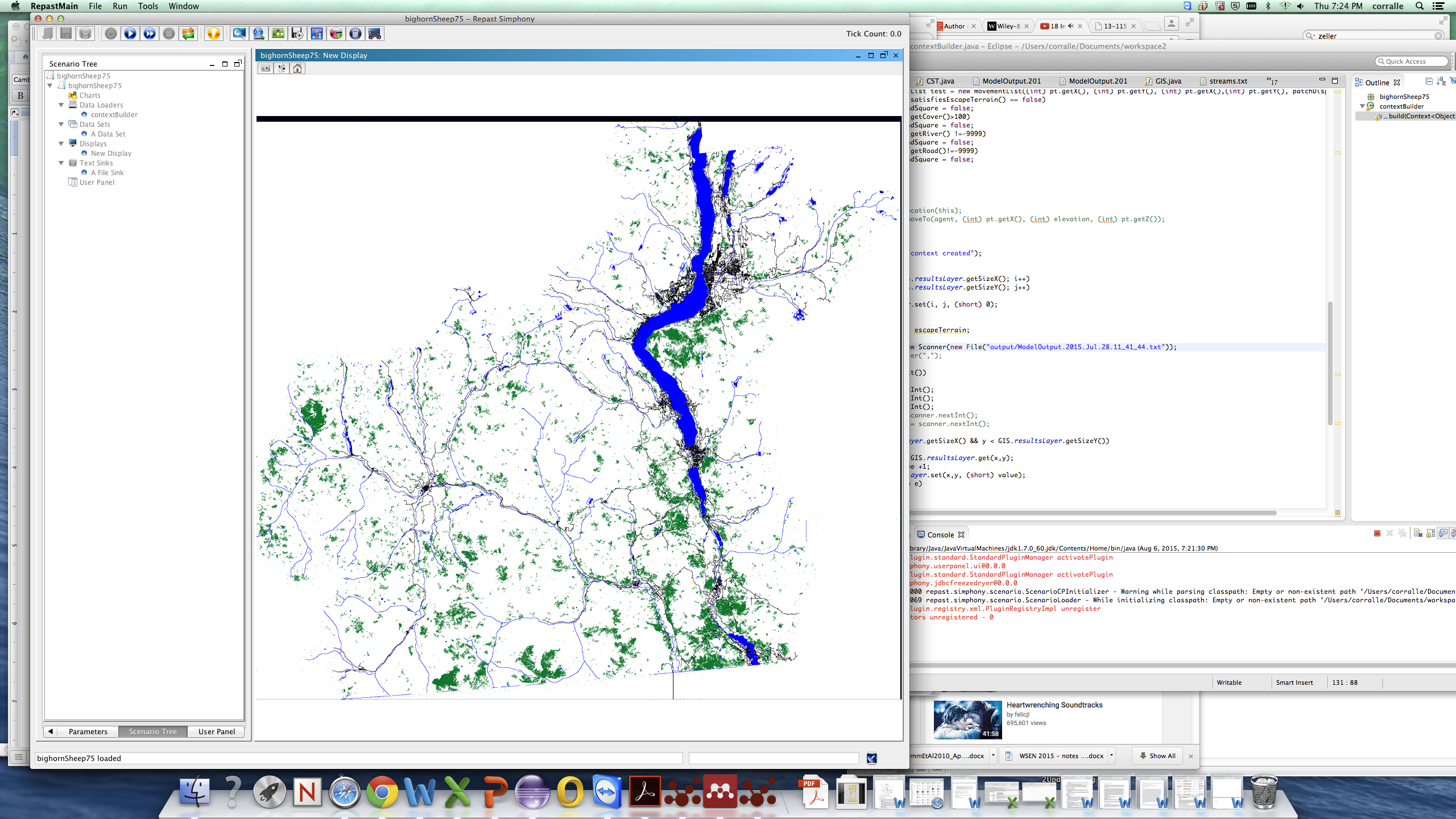

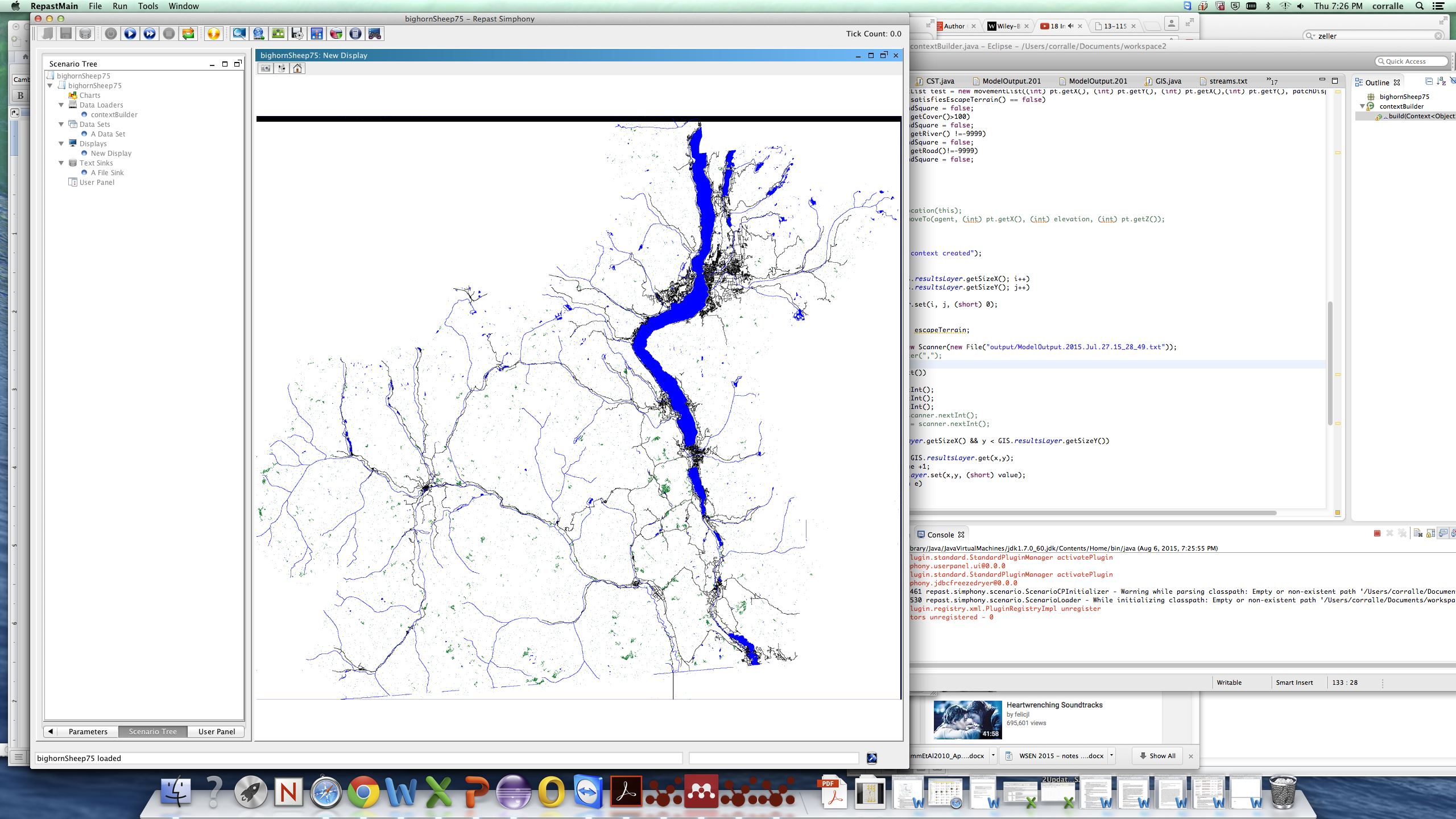

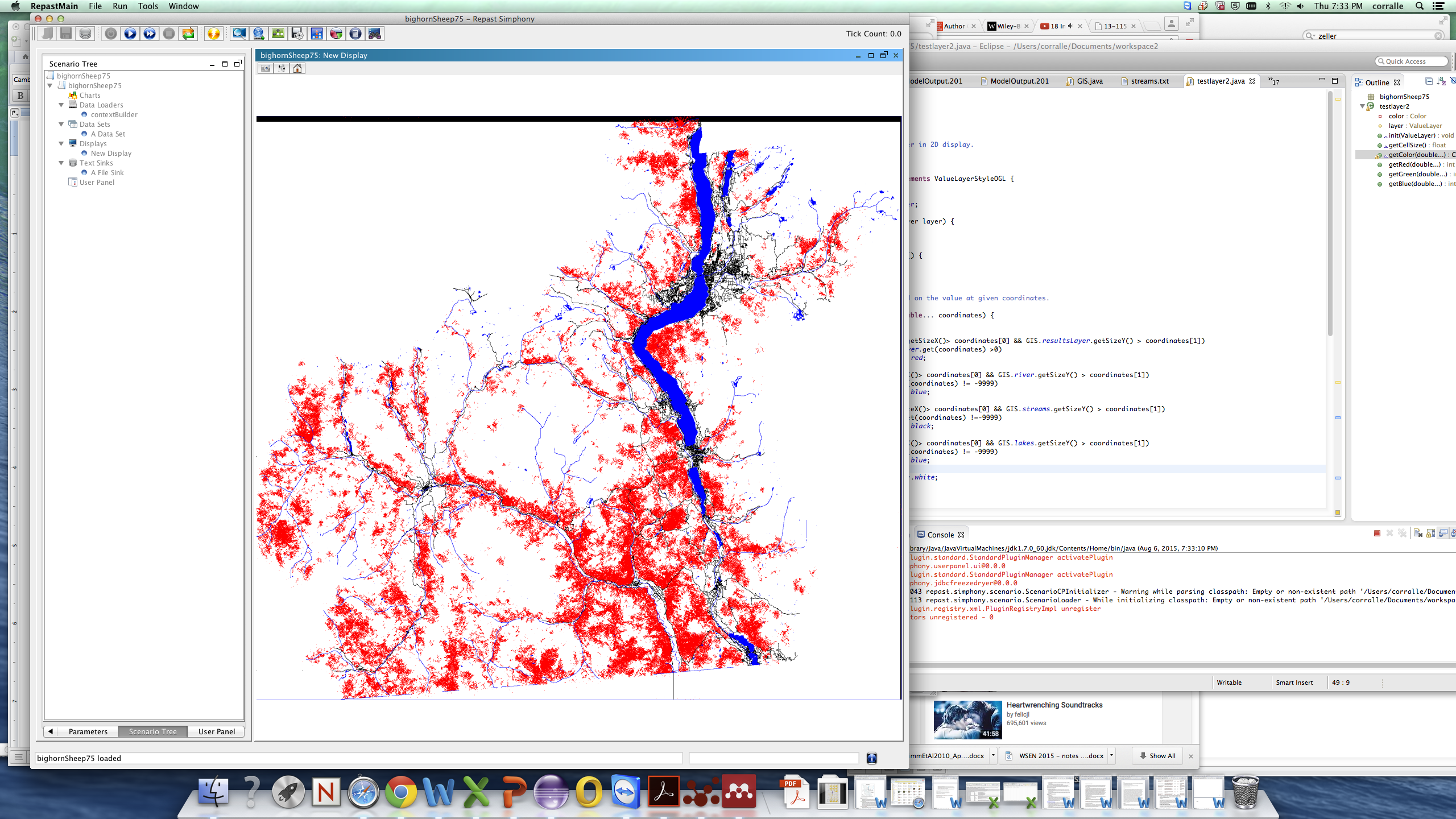

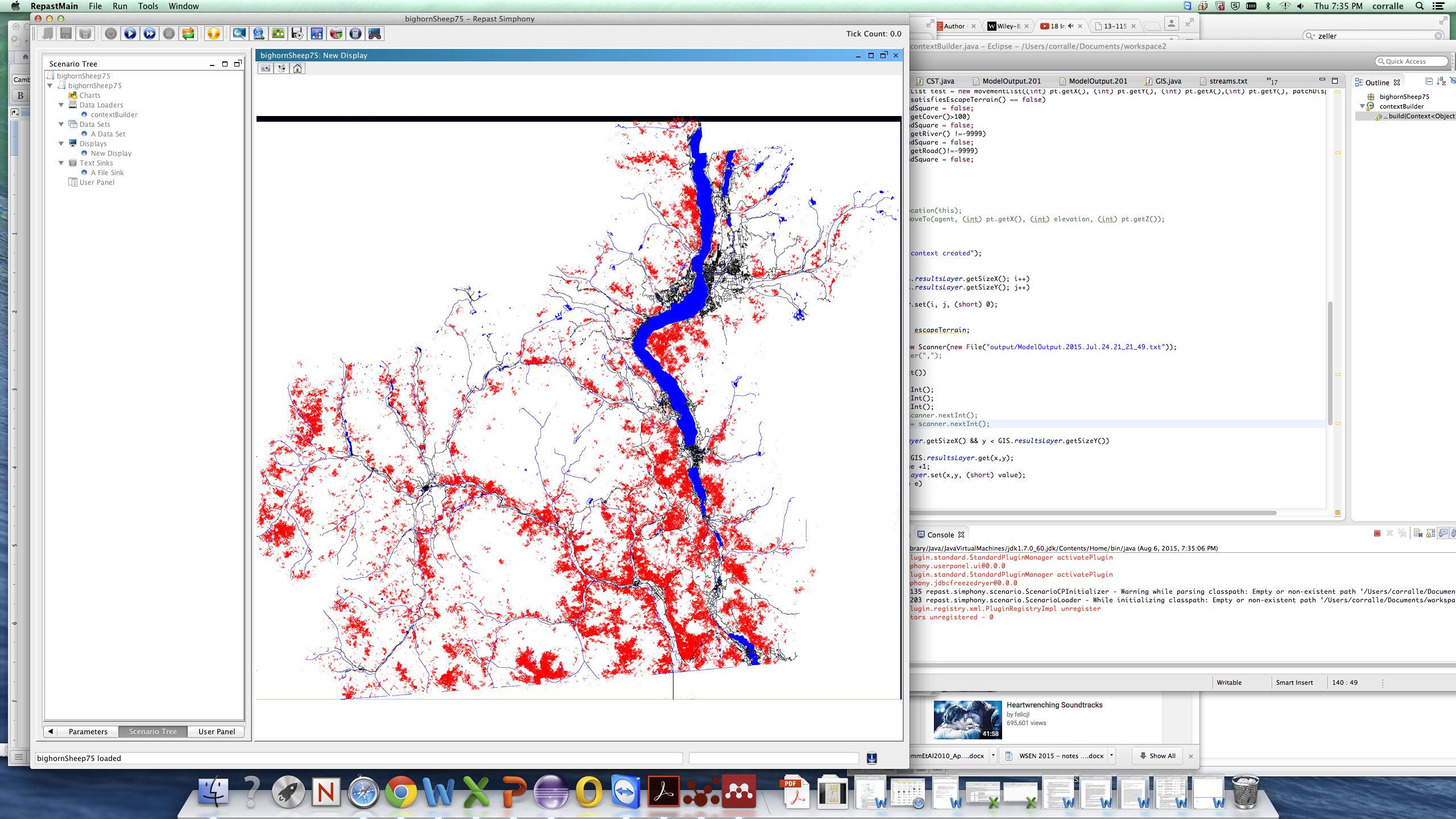

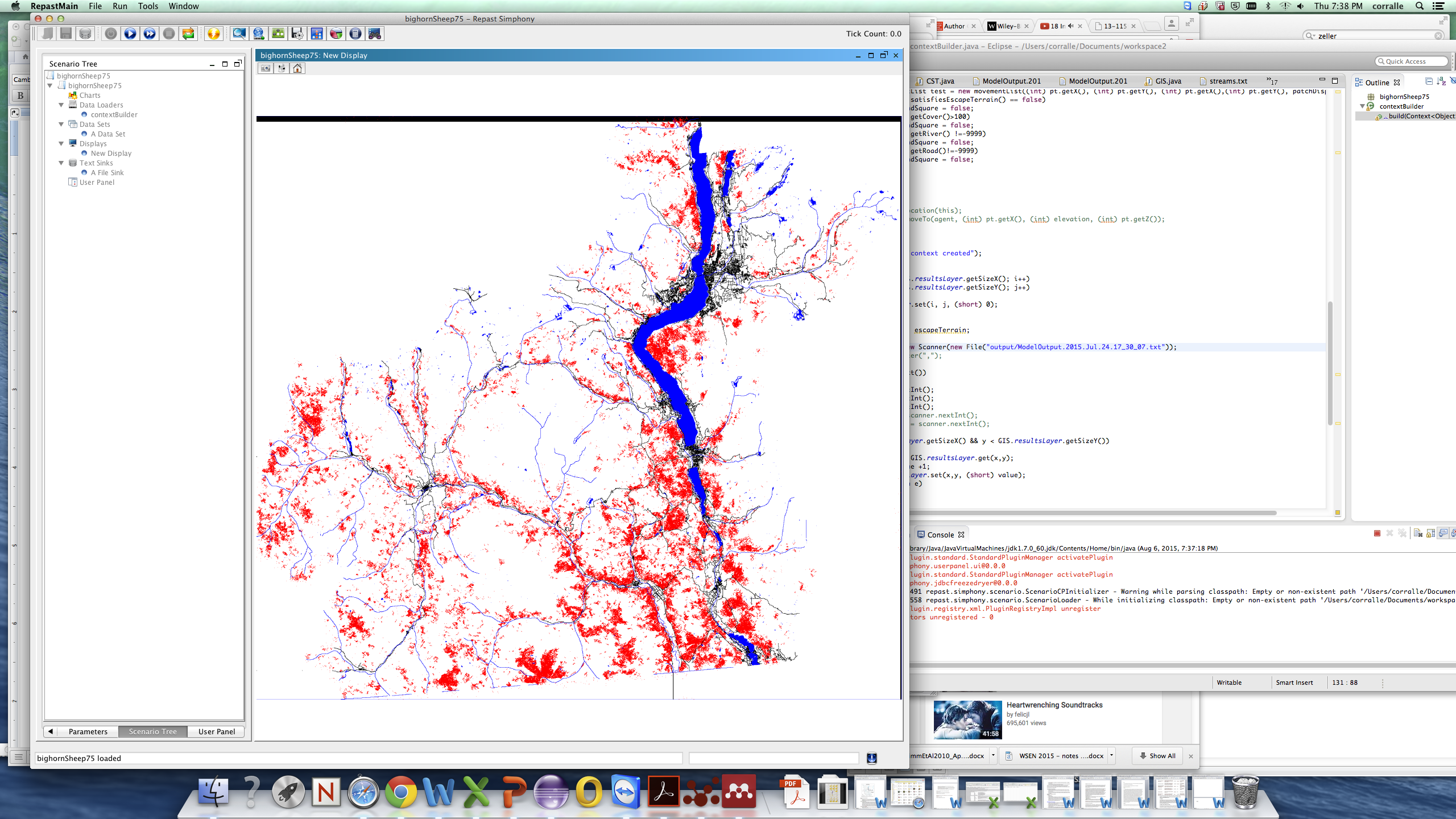

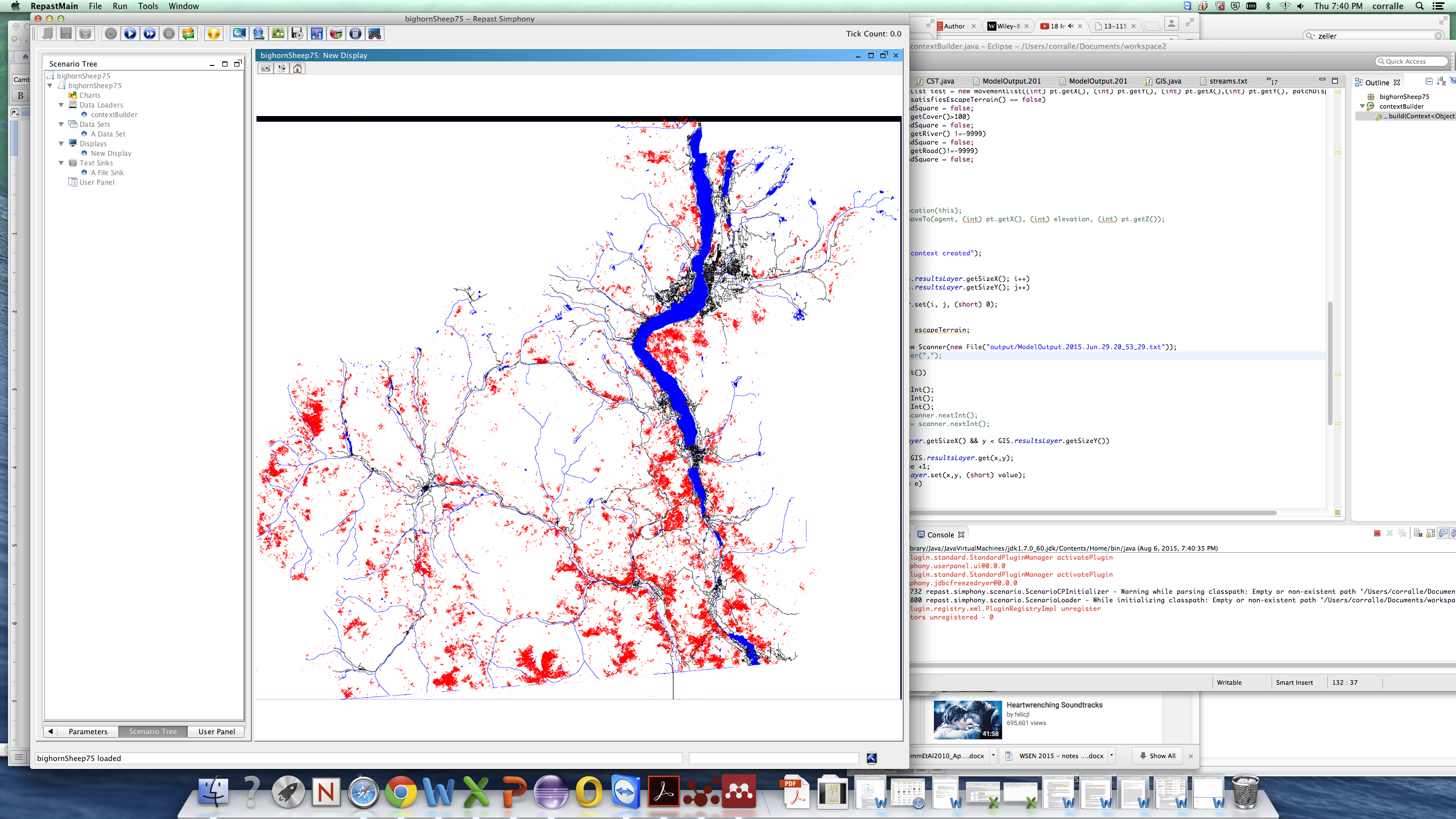

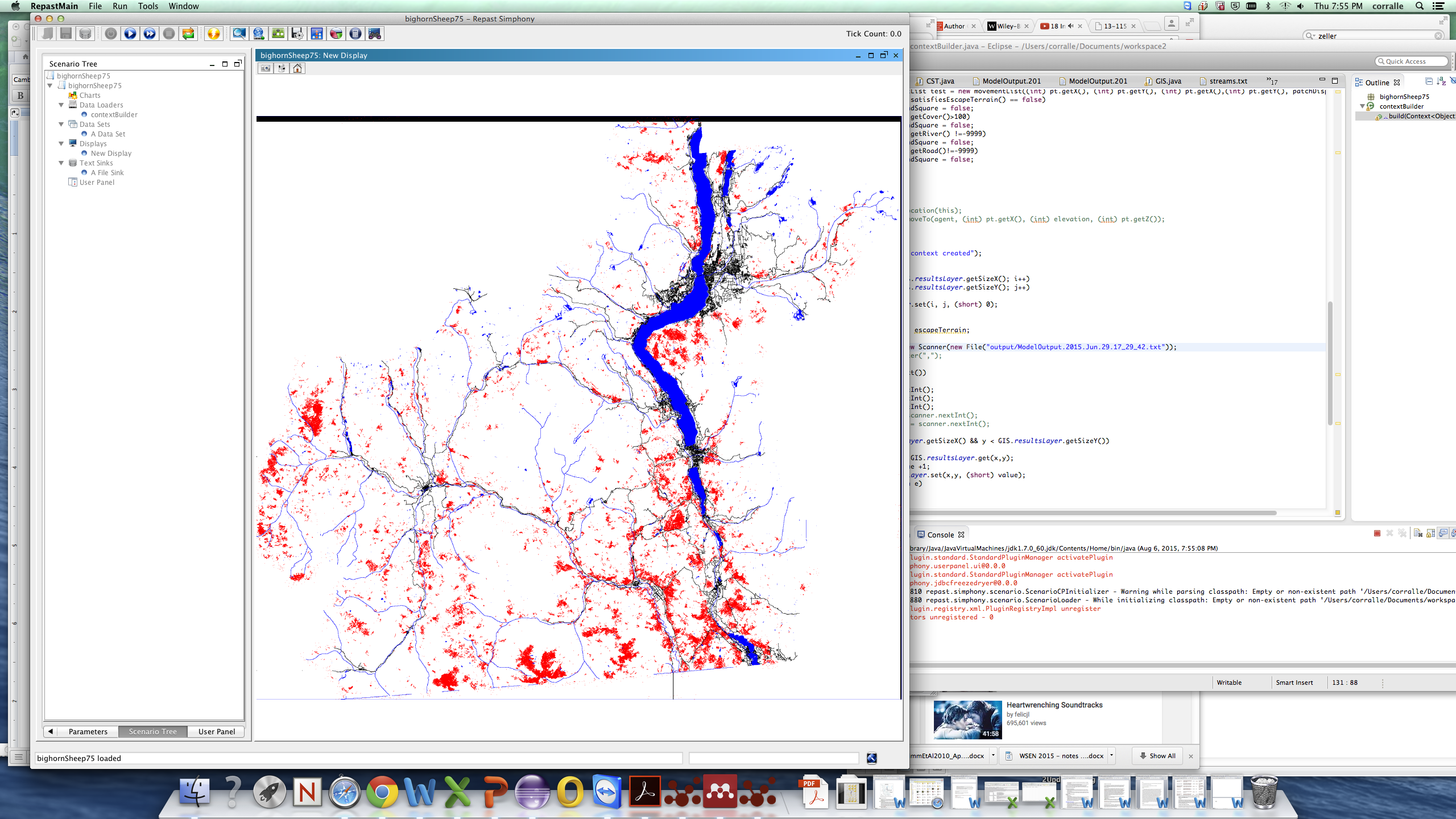

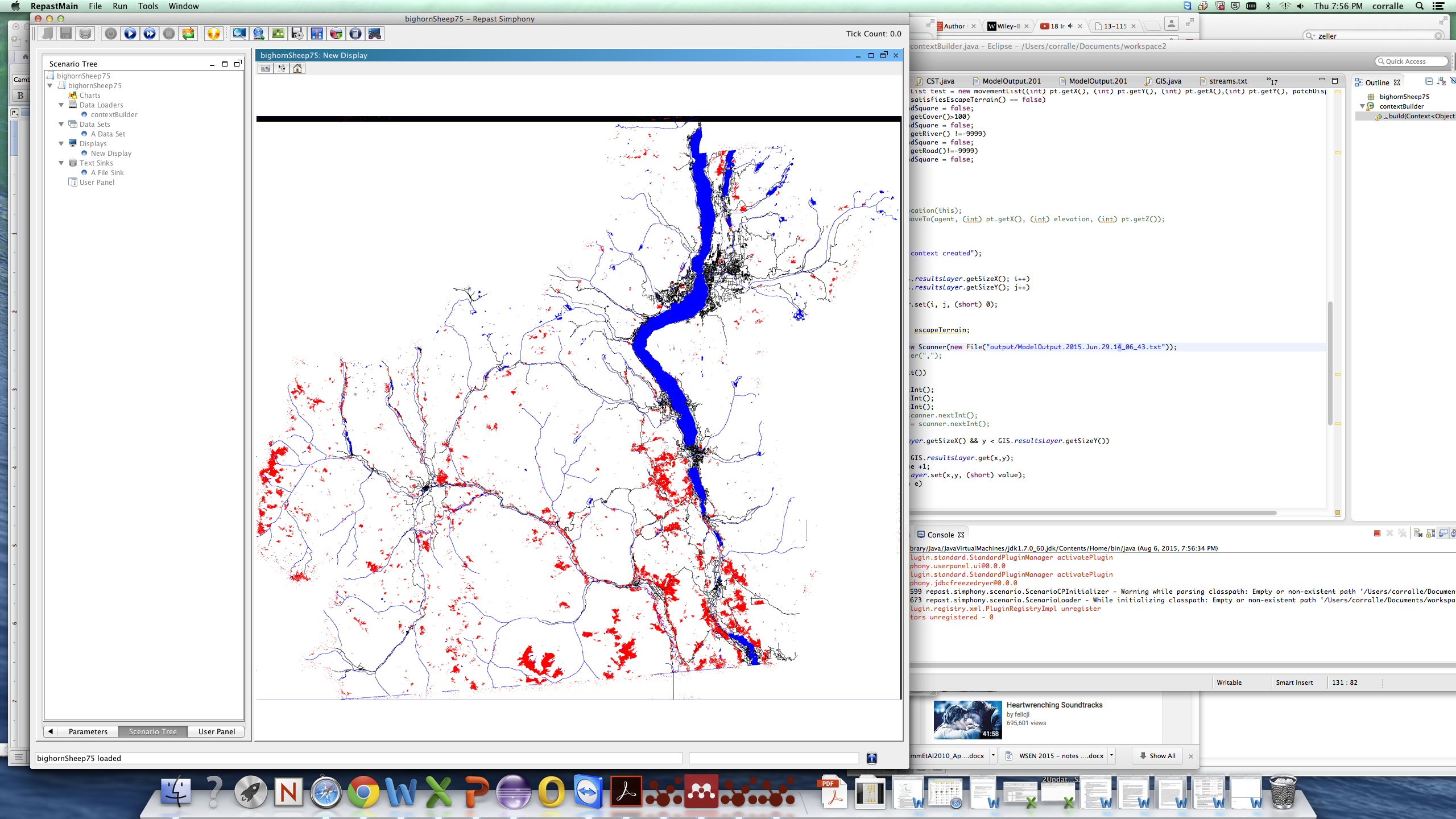


Figure 1. Individual based model sensitivity analysis. The value of escape terrain (E.T.) and crown cover (C.C.) simulated bighorn sheep responded to was varied and the resulting connectivity map displayed. Crown cover < 40% and escape terrain > 40 degrees was used in the final model (outlined in a square).

E.T. > 20

E.T. > 30

E.T. > 40

C.C. < 60 C.C. < 50 C.C. < 40 C.C. < 30 C.C. < 20 C.C. < 0

**References**

DeCesare NJ., Pletscher DH. 2006. Movements, connectivity, and resource selection of rocky mountain bighorn sheep. *Journal of Mammalogy* *87*:531–538.

Demarchi RA., Hartwig CL., & Demarchi DA. 2000. Status of the California Bighorn Sheep in British Columbia. *BC Ministry Environment, Lands and Parks, Wildlife Branch, Victoria, BC. Wildllife Bulletin No. B-98:* 1-53.

Epps CW., Palsbøll PJ., Wehausen JD., Roderick GK., Ramey RR., McCullough DR. 2005. Highways block gene flow and cause a rapid decline in genetic diversity of desert bighorn sheep. *Ecology Letters* 8:1029–1038.

MacArthur RA., Johnston RH., Geist V. 1979. Factors influencing heart rate in free-ranging bighorn sheep: a physiological approach to the study of wildlife harassment. *Canadian Journal of Zoology* 57:2010–2021.

Shannon NH., Hudson RJ., Brink VC., Kitts WD. 1975. Determinants of spatial distribution of rocky mountain bighorn sheep. *The Journal of Wildlife Management* 39:387–401.

Smith T., Flinders J., & Winn D. 1991. A habitat evaluation procedure for rocky mountain bighon sheep in the intermountain west. *The Great Basin Naturalists* 51:205-225.

Tilton ME., Willard EE. 1982. Winter habitat selection by mountain sheep. *The Journal of Wildlife Management 46:*359-366.

Warman L., Robertson S., Haney A., Sarell M. 1998. Habitat capability and suitability models for 34 wildlife species using terrestrial ecosystem mapping in the South Okanagan and Lower Similkameen Study area and forest cover mapping in the Penticton forest district. Prepared for the Wildlife Branch, Ministry of Environment, Lands and Parks, Penticton, B.C.
