# Supplementary material for: An individual-based modelling approach to estimate landscape connectivity for bighorn sheep (Ovis canadensis)
Source: PeerJ. 2016 May 5;4:e2001. doi: 10.7717/peerj.2001 (PMC4860333; doi:10.7717/peerj.2001)
Supplement: Table S1 — This table lists the geospatial data layers used as input to the model, and the sources of these data. [file peerj-04-2001-s002.docx]

**Supplemental Table S1: Geospatial input data**

Table S1. Source data layers used to create and parameterize models

| *Dataset name* | *Description* | *Scale* | *Date* | *Source* |
| --- | --- | --- | --- | --- |
| Vegetation Resource Inventory (VRI) | Detailed information related to composition of habitat and non-habitat in British Columbia | 1:20,000 | Unknown | B.C. Land and Resource Data Warehouse |
| Digital Elevation Model | Elevation model of study area. Derived from the B.C. Terrain Resource Information Mapping (TRIM) data. | 1:250,000 | Unknown | B.C. Land and Resource Data Warehouse |
| Digital Road Atlas (DRA) – Demographic Partially-Attributed Roads | Provides publically accessible information on roads in British Columbia | Unknown | 2014 | Ministry of Forests, Lands and Natural Resource Operations, GeoBC |
| Freshwater Atlas | Hydrological features for the Okanagan. We extracted vector data on lakes and rivers from this layer. | 1:20,000 | 2010 | B.C. Land and Resource Data Warehouse |
| CanMap Streetfiles | Patterns of human land use transformation in BC | Unknown | 2011 | DMTI Spatial, Inc. |
| Bighorn Sheep Occurrence points* | A derived layer combining multiple bighorn sheep occurrence points | Varies | 1968-2012 | Ministry of Forests, Lands and Natural Resource Operations |

*****We created this layer by combing multiple ungulate inventory studies in the Okanagan Valley.
